# Does the type of oral anticoagulant matter for stroke prevention or bleeding in patients with atrial fibrillation after cardiac surgery? A systematic review and meta-analysis

**DOI:** 10.1093/ehjopen/oeaf062

**Published:** 2025-06-04

**Authors:** Marc M Terpstra, Tim A C de Vries, Ellis Oortwijn, Rob A F de Lind van Wijngaarden, Joris R de Groot

**Affiliations:** Department of Clinical and Experimental Cardiology, Heart Center, Amsterdam University Medical Centers, University of Amsterdam, Meibergdreef 9, 1105 AZ Amsterdam, The Netherlands; Amsterdam Cardiovascular Sciences, Heart Failure and Arrhythmias, Meibergdreef 9, 1105AZ Amsterdam, The Netherlands; Department of Clinical and Experimental Cardiology, Heart Center, Amsterdam University Medical Centers, University of Amsterdam, Meibergdreef 9, 1105 AZ Amsterdam, The Netherlands; Amsterdam Cardiovascular Sciences, Heart Failure and Arrhythmias, Meibergdreef 9, 1105AZ Amsterdam, The Netherlands; Department of Cardiology, Rijnstate Hospital, Wagnerlaan 55, 6815 AD Arnhem, The Netherlands; Department of Clinical Geriatrics, Radboud University Medical Centre, Geert Grooteplein Zuid 10, 6525 GA Nijmegen, The Netherlands; Department of Clinical and Experimental Cardiology, Heart Center, Amsterdam University Medical Centers, University of Amsterdam, Meibergdreef 9, 1105 AZ Amsterdam, The Netherlands; Amsterdam Cardiovascular Sciences, Heart Failure and Arrhythmias, Meibergdreef 9, 1105AZ Amsterdam, The Netherlands; Amsterdam Cardiovascular Sciences, Heart Failure and Arrhythmias, Meibergdreef 9, 1105AZ Amsterdam, The Netherlands; Department of Cardiothoracic Surgery, Heart Center, Amsterdam University Medical Centers, Meibergdreef 9, 1105 AZ Amsterdam, The Netherlands; Department of Clinical and Experimental Cardiology, Heart Center, Amsterdam University Medical Centers, University of Amsterdam, Meibergdreef 9, 1105 AZ Amsterdam, The Netherlands; Amsterdam Cardiovascular Sciences, Heart Failure and Arrhythmias, Meibergdreef 9, 1105AZ Amsterdam, The Netherlands

**Keywords:** Atrial fibrillation, Cardiac surgery, Anticoagulation, Stroke prevention, Meta-analysis

## Abstract

**Aims:**

Unlike in non-surgical settings, many centres continue to favour vitamin K antagonists (VKAs) for stroke prevention in atrial fibrillation (AF) following major cardiac surgery. Current guidelines indicate insufficient data on the use of direct oral anticoagulants (DOACs) early after cardiac surgery. This study aims to evaluate whether DOACs are non-inferior to VKAs in terms of efficacy and safety for stroke prevention in post-operative AF.

**Methods and results:**

MEDLINE, EMBASE, CENTRAL, and Clinicaltrials.gov were searched from inception till 2 July 2024, and relevant reviews were screened as grey literature. Studies comparing DOACs with VKAs for stroke prevention in patients with (post-operative) AF after major cardiac surgery were included. Studies on patients with mechanical valve replacement or moderate to severe mitral stenosis were excluded. Outcomes of interest included thromboembolic events, major bleeding and mortality up to 6 months after cardiac surgery. Eleven studies, including two randomized controlled trials, reporting on >18,000 patients were analyzed. There were no significant differences between DOACs and VKAs in thromboembolic events (OR: 0.96; CI: 0.62–1.50; I^2^: 0%), any stroke (OR: 1.44; CI: 0.61–3.41; I^2^: 0%), major bleeding (OR: 0.97; CI: 0.60–1.56; I^2^: 48%), all-cause mortality (OR: 1.00; CI: 0.73–1.37; I^2^: 0%) or admission duration (MD: −0.33; CI: −1.16–0.49; I^2^: 0%) in the first 6 months after cardiac surgery.

**Conclusion:**

There is no high-quality evidence that DOACs and VKAs differ in efficacy or safety for stroke prevention in AF after cardiac surgery. While awaiting high-quality randomized data, our meta-analysis found no evidence to support routinely avoiding DOACs or favouring VKAs in this setting.

**Registration:**

Review registration number: CRD42023412592.

## Graphical Abstract

**Graphical Abstract:**
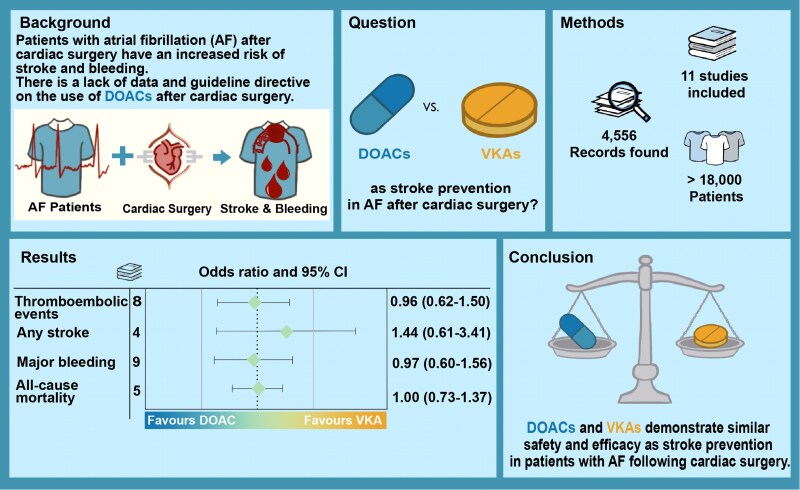

## Introduction

Atrial fibrillation (AF) is a common arrhythmia, affecting one in three people during their lifetime, and increasing the risk of stroke and death. Patients with AF have approximately a five-fold higher risk of stroke compared with adults without this arrhythmia. To prevent thrombus formation and stroke, oral anticoagulation (OAC) is generally recommended. However, the use of OACs is not without downsides, as they increase the risk of bleeding.^1–3^

The introduction of direct oral anticoagulants (DOACs) has greatly simplified anticoagulant treatment because, unlike vitamin K antagonists (VKAs), they can be given in fixed doses and do not require routine monitoring of anticoagulant intensity or perioperative bridging. Besides their better convenience, large randomized trials have demonstrated that DOACs are at least as effective as VKAs, but generally lead to less bleeding and are associated with reduced mortality.^4,5^ Cardiac surgery increases risks of both bleeding and thrombosis. In addition, post-operative bleeding events may necessitate reintervention, potentially requiring reversal of anticoagulant activity, which in turn increases the thrombotic risk. Given these competing risks, the optimal anticoagulation strategy in the post-operative setting remains uncertain. This complexity has historically led to a preference for VKAs in the early post-operative setting, due to presumed better safety of VKAs after surgery based on easier monitoring, clear reversal strategies, and the lack of sufficient data on DOACs safety in this context. Although DOACs are being increasingly prescribed after cardiac surgery, VKAs remain the predominant OAC after cardiac surgery in many centres.^6–12^

Recent guideline recommendations reflect this uncertainty, acknowledging that DOACs may be a valid alternative to VKAs in various AF settings, but they do not provide specific recommendations for DOAC use in the first months after surgery. For example, the EACTS guidelines suggest DOACs may be considered after three months in patients with bioprosthetic valves and a pre-existing indication for anticoagulation, but offer no recommendation for earlier initiation. Regarding post-operative AF (POAF), guidelines note that long-term OAC may be considered in high-risk patients, but again offer limited guidance on agent selection or timing in the immediate post-operative window. Importantly, the trials that have informed these guidelines—such as RIVER and ENAVLE—included only small numbers of patients in the early post-operative phase, which helps explain the absence of strong, evidence-based recommendations during this critical period.^13–18^

Furthermore, in centres where VKAs are preferred over DOACs in the early post-operative period, this may involve initiating VKAs in patients who were previously on DOACs, followed by a subsequent switch back to DOACs. This practice is unsupported by evidence, and switching OAC agents has been associated with increased risks of both bleeding and thromboembolic events during the transition period.^19,20^

Two previous reviews have examined the use of DOACs vs. VKAs for stroke prevention in AF after cardiac interventions. Hage *et al*.^21^ analysed a mixed cohort of patients following percutaneous or surgical cardiac procedures and found that DOACs were associated with a lower risk of stroke while demonstrating a comparable safety profile to VKAs. Koh *et al*.^22^ pooled studies of patients with AF and prior cardiac surgery but did not focus specifically on the early post-operative phase, and many included patients were anticoagulated long after surgery. As such, evidence directly addressing the early post-operative period after cardiac surgery remains scarce.^21,22^

To address this evidence gap and guide clinicians in their choice of anticoagulant strategy, we conducted a systematic review and meta-analysis comparing the efficacy and safety of DOACs compared with VKAs, in AF patients early after cardiothoracic surgery. We included both patients with pre-existing AF as those developing POAF. Of note, in this analysis we focus on the comparison between VKAs and DOACs, and do not take a stand on whether or not OAC is indicated, for example in the setting of POAF. Patients with a clear indication for VKAs (i.e. with mechanical heart valves or a moderate to severe mitral stenosis) were excluded. We hypothesized that DOACs have similar safety and efficacy compared with VKAs in this setting.

## Methods

This study adhered to our registered protocol, Prospero (CRD42023412592), and the PRISMA statement presented in Supplementary material online, Appendix  *A*.^23^

### Search strategy

A comprehensive search strategy was developed in collaboration with a medical librarian (AM) and involved searching: MEDLINE via OVID, Embase via OVID, Central and clinicaltrials.gov from inception till 2 July 2024. The strategy included terms relating to AF, cardiac surgery, DOACs and VKAs. We applied no language restrictions to the search. The search strategies for MEDLINE and EMBASE are shown in Supplementary material online, *Appendix B*. Systematic reviews on similar topics and reference lists of included studies were screened for additional eligible references.

### Study selection

Studies comparing DOACs and VKAs for stroke prevention in patients with AF or atrial flutter undergoing cardiothoracic surgeries were included. Eligible studies reported on pre-existing AF or POAF and presented data on a primary outcome. Studies concentrating on contraindications to the medications, non-cardiac surgical procedures, or patients with a different indication for treatment with an anticoagulant (such as the presence of a mechanical valve prosthesis or moderate to severe mitral stenosis) were excluded. Eligible study designs were randomized controlled trials (RCTs), cohort studies and case-control studies. The full list of our eligibility criteria is specified in Supplementary material online, *Appendix C*.

After removing duplicate records, two reviewers (MT and EO) independently screened titles and abstracts. Full texts of potentially eligible reports were reviewed, and disagreements were resolved through discussion or through involving a third author. Decisions and disagreements were documented using the Rayyan tool and the study selection process was depicted using a PRISMA flow diagram.^23,24^

### Outcomes

The primary outcomes were thromboembolic events, major bleeding, all-cause mortality, any stroke, reintervention, readmission and admission duration. Secondary outcomes included clinically relevant non-major bleeding (CRNM), ischaemic stroke, haemorrhagic stroke and systemic embolisms. Outcomes were classified into 6 and 12-month follow-up periods, with primary analyses focusing on events occurring within the first 6 months following OAC therapy initiation.

### Data extraction

Two independent reviewers (MT and TdV) extracted data from eligible studies using SRDR+ electronic pre-piloted forms.^25^ These data included methodological characteristics, study population demographics and outcomes. Divergent extractions were discussed and resolved between the two reviewers. We sought missing data by reviewing study protocols or contacting authors.

### Risk of bias and quality assessment

Bias and quality were assessed in accordance with our pre-specified criteria (see Supplementary material online, *Appendix D*). Bias assessments were performed in duplicate by two independent reviewers (MT and TdV), and discrepancies were discussed between these reviewers.

### Data synthesis

Meta-analysis was conducted when at least two eligible studies reported on the outcome. Due to heterogeneity in follow-up durations, we classified data ranging from 4 to 6 weeks as one month. In addition, we pooled index dates ranging from surgery to the start of OAC treatment within three months. Initially, we generated separate forest plots for RCTs and non-randomized studies (NRS) to assess consistency. Observing similar effect directions across both study types, we proceeded with pooled analyses, consistent with prior methodological recommendations.^26^

In our primary analyses, we combined data associated with AF and POAF, pooled all surgical interventions and combined factor IIa inhibitors and factor Xa inhibitors as DOACs. Subsequently, we conducted meta-regression and subgroup analyses to explore the impact of individual subgroups, focusing on study design (RCT vs. NRS), surgery type (isolated valve surgery vs. isolated CABG vs. other procedures) and AF type (pre-existing AF vs. POAF vs. combined).

### Statistical analysis

Dersimonian and Laird random effects models were used to pool data.^27^ Statistical analyses were performed using R (version 4.3.2), with the packages metafor and metamedian.^28–30^ Pooled log odds ratios were calculated from event rates and transformed accordingly, with a 0.5 continuity correction applied when no events were observed in an arm. For continuous outcomes, we used the quantile estimation method by Mcgrath *et al*.^31^ to pool means and medians. Two-sided *P*-values <0.05 were considered statistically significant. Outcomes are presented as event rates and odds ratios (OR) with 95% confidence intervals (CI); means with standard deviation (SD), medians with 25th and 75th percentiles or mean differences (MD) with 95% CI. Forest plots visualize the outcomes.

Sensitivity analyses were conducted to evaluate the robustness of our main findings. These analyses focused on the impacts of pooling decisions, risk of bias, inclusion of multiple OAC indications, and inclusion of studies with unclear or slightly different outcome definitions.^32^ Our sensitivity analyses are further elaborated in *Table 1*.

**Table 1:** Characteristics of included studies

Study	Enroll. period	Design	Key inclusion criteria	Key exclusion criteria	Study arm	*n* Patients	Study medication	Age in years	Males (%)	*N* Pre-existing AF	*N* POAF
Anderson *et al*.^33,a^	01/2013–03/2015	Single centre RCT	CABG POAF	Pre-existing AF	DOAC	27	Apixaban dabigatran rivaroxaban	73.1 ± 6.8	22 (82%)	—	27
					VKA	45	Warfarin	73.2 ± 8.2	37 (82%)	—	45
Brochu *et al*.^34^	01/2019–03/2021	Single centre retrospective cohort	Cardiac surgery POAF	TransCath procedure	DOAC	94	apixaban dabigatran edoxaban rivaroxaban	71 ± 10	85 (90%)	44	50
					VKA	139	Warfarin	70 ± 9	106 (76%)	69	70
Chapin *et al*.^32,b^	09/2016–05/2019	Single cent. RCT	CABG POAF	Stroke ≤ 7 days.	DOAC	28	Apixaban	71 ± 8.1	25 (89%)	—	28
					VKA	28	Warfarin	69.5 ± 7.5	20 (71%)	—	28
Guimaraes *et al*.^16,c,d^	04/2016–07/2019	Multicent. RCT	Mitral valve -surgery AF	Transient POAF.	DOAC	94	Rivaroxaban	Study total: 59.4 ± 2.4	Study total: 189 (38%)	Study total: 500	—
					VKA	95	Warfarin	Study total: 59.2 ± 11.8	209 (41%)	Study total: 505	
Moser *et al*.^35^	07/2014–06/2021	Multicent. retrospect. Cohort	Valve surgery AF	TransCath procedure & Death during admission	DOAC	570	Apixaban dabigatran edoxaban rivaroxaban	71 ± 8	384 (67%)	296	274
					VKA	1173	Warfarin	68 ± 10	759 (65%)	435	738
Nauffal *et al*.^36^	07/2017–12/2018	National retrospect. cohort	CABG/valve surgery & POAF	Preexist. AF & Death/TE/MB during admission	DOAC	7135	Unspecified DOACs	69.4 ± 8.5	5307 (74%)	−	7135
					VKA	7135	Warfarin	69.5 ± 8.6	5311 (74%)	—	7135
Pasciolla *et al*.^37,e^	01/2014–06/2018	Single cent. retrospect. cohort	Valve surgery	Switching or stopping within 6 months	DOAC	119	Apixaban dabigatran edoxaban rivaroxaban	71.9 ± 9.19	72 (57%)	57	62
					VKA	65	Warfarin	74.5 ± 9.39	39 (56%)	45	19
Piepiorka-Broniecka *et al*.^38^	NR	Single cent. RCT	Valve surgery AF/POAF		DOAC	25	Apixaban	65.9 ± 8.6	14 (56%)	6	19
					VKA	25	Warfarin	68.2 ± 6.5	13 (52%)	7	18
Shim *et al*.^17,c,d^	12/2017–09/2019	Single cent. RCT	Valve surgery	TransCath procedure & MB/TE days after surgery	DOAC	65	Edoxaban	Study total: 67.0 ± 12.3	Study total: 52 (48%)	65	0
					VKA	67	warfarin	Study total: 67.7 ± 10.0	Study total: 62 (57%)	67	0
Skogseid *et al*.^39^	01/2010–12/2016	National retrospect. Cohort	Valve surgery	Death during admission	DOAC	164	Apixaban dabigatran rivaroxaban	Study total: 76 (70–82)	Study total: 220 (63%)	NR	NR
					VKA	1078	Warfarin	Study total: 74 (67–79)	Study total: 1413 (68%)	NR	NR
Woldendorp *et al*.^40,c^	01/2015–12/2018	Single cent. retrospect. cohort	CABG	TransCath procedure	DOAC	29	Apixaban dabigatran rivaroxaban	Study total: 64.5 ± 10.5	Study total: 797 (83%)	—	29
					VKA	77	Warfarin			—	77

NR, Not reported.

^a^Missing SD for admission duration outcome. Bleeding defined as CRNM after deliberation.

^b^Unclear major bleeding definition, sensitivity analysis performed.

^c^Only subgroup that received anticoagulation was relevant.

^d^Subgroup of RCT treated as non-randomized.

^e^92% of population had AF as indication. All events occurred in AF patients. Study included and sensitivity analysis performed.

Funnel plots were created to assess publication bias. Between-study heterogeneity was evaluated using prediction intervals, the Chi-squared test and the I^2^ statistic, with classification as low (<30%), moderate (30–59%) or high (≥60%). We further investigated heterogeneity in our summary estimates through pre-specified subgroup analyses based on the type of AF, type of surgery, and study design. These subgroup analyses were limited to thromboembolic events and major bleeding outcomes, as the remaining primary outcomes did not have a sufficient number of included studies to provide meaningful insights. Additionally, due to a lack of outcome data stratified per type of DOAC, we were unable to perform subgroup analyses based on the type of DOAC. To further address residual heterogeneity, we performed exploratory *post hoc* meta-regression analyses focusing on our pre-specified subgroups. Given the limited number of studies included in these analyses, the meta-regression results are inherently exploratory and should be interpreted with caution. Meta-regression results are presented as regression coefficients with 95% CI, *P*-values, and the proportion of variance explained with *R*^2^.

## Results

### Study selection

Our search strategy yielded 5194 hits. After deduplication, a total of 4557 records were identified. After screening titles and abstracts, we selected 127 reports for full-text assessment, and of these, 11 reports were included in our final analysis. The process of study selection is depicted in Supplementary material online, *Appendix F*.

### Study characteristics and bias assessment

The characteristics of the included studies are summarized in *Table 1*. We included eleven studies, comprising seven retrospective cohort studies and four RCTs.^16,17,32–40^ However, we considered two of the RCTs as non-randomized since only the populations within subgroup analyses were relevant to our question.^16^  ^,17^ Most studies (4 out of 11) focused on patients with POAF, while two studied patients with AF, and five examined a mixture of AF and POAF patients. The most common surgical interventions were valve replacements, valve repairs and coronary artery bypass grafting (CABG). OAC was typically initiated during admission following surgery in the primary analyses, although a subset of patients in two studies may have started treatment up to 3 months following surgery.^35,39^ Warfarin was the only VKA used, while all four DOACs were employed in the DOAC arm. In Supplementary material online, *Appendix E*, we present the funding and conflicts of included studies and the results of our risk of bias and quality assessments.

### Thromboembolic events and any stroke

**Figure 1:**
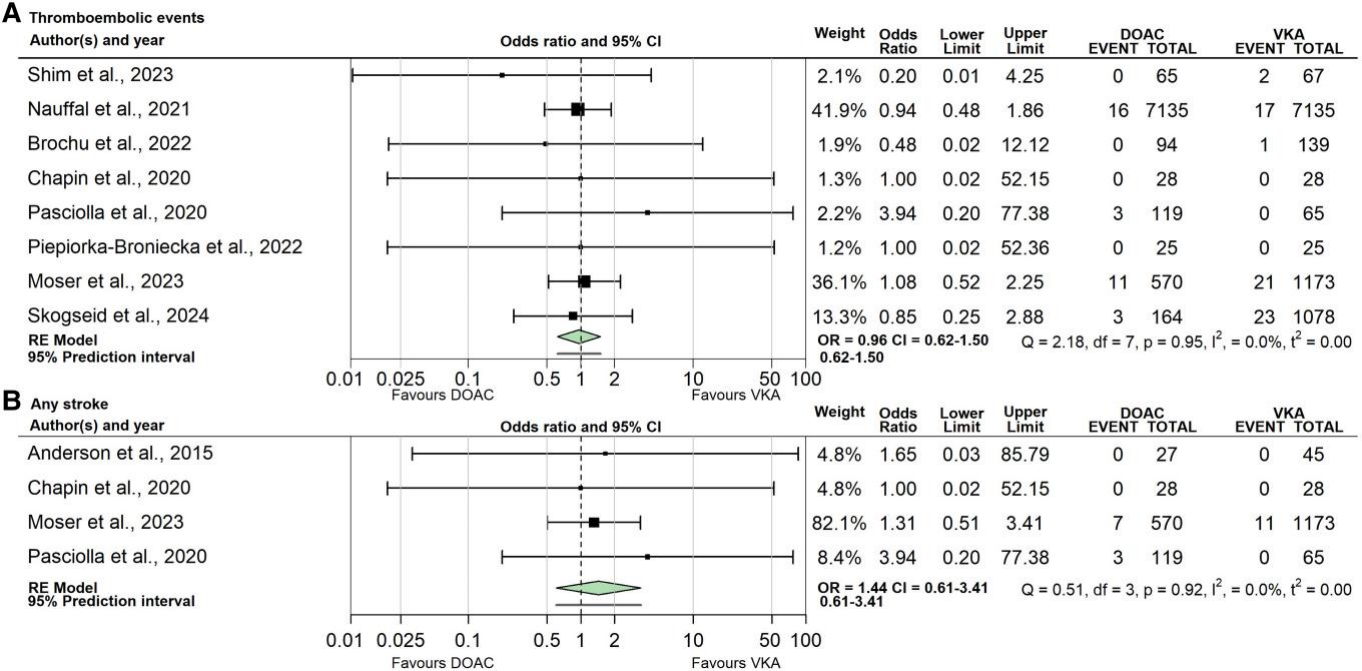
(*A*) Thromboembolic events up to 6 months after anticoagulation initiation. (*B*) Any stroke up to 6 months after anticoagulation initiation. OR, odds ratio; CI, Confidence interval; Q, Q statistic; Df, Degrees of freedom; *P*, *P*-value; I^2^, Proportion of between-study heterogeneity; *t*^2^, Between-study variance estimate.

*Figure 1* illustrates the relative effect of DOAC vs. VKA therapy on thromboembolic events (Panel A) and any stroke (Panel B). Eight studies comprising 17 910 patients reported on thromboembolic events up to 6 months after starting anticoagulation. There were 33 reported thromboembolic events in patients given a DOAC compared with 64 in those treated with a VKA (OR: 0.96, CI: 0.62–1.50, I^2^: 0%). Risk of bias was rated critical in one study in this analysis, high in five and moderate in two. Level of evidence for this outcome was graded as very low, due to serious risk of bias and imprecision.

Four studies comprising 2055 patients reported on any stroke up to 6 months after starting anticoagulation. There were 10 strokes in patients given a DOAC compared with 11 in those treated with a VKA (OR: 1.44, CI: 0.61–3.41, I^2^: 0%). Risk of bias was rated as critical in one study in this analysis, high in two and moderate in two. Level of evidence for this outcome was graded as very low, due to very serious risk of bias and serious imprecision.

### Major bleeding and all-cause mortality

**Figure 2:**
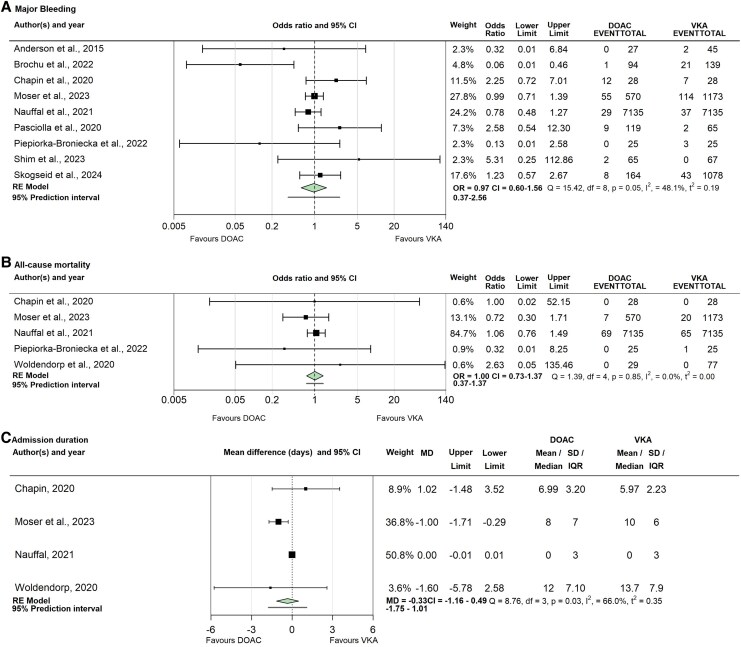
(*A*) Major bleeding events up to 6 months after anticoagulation initiation. (*B*) All-cause mortality up to 6 months after anticoagulation. (*C*) Admission duration in days. CI, Confidence interval; Df, Degrees of freedom; I^2^, Proportion of between-study heterogeneity; IQR, Interquartile range; MD, Mean difference; OR, odds ratio; *P*, *P*-value; Q, Q statistic; SD, Standard deviation; *t*^2^, Between-study variance estimate.

*Figure 2* illustrates the relative effect of DOAC vs. VKA therapy on major bleeding (Panel A) and all-cause mortality (Panel B). Nine studies comprising 17 982 patients reported on major bleeding up to 6 months after starting anticoagulation. There were 116 major bleeding events in patients given a DOAC compared with 229 in those treated with a VKA (OR: 0.97, CI: 0.60–1.56, I^2^: 48.1%). Risk of bias was rated as critical in one study in this analysis, high in five and moderate in two. Level of evidence for this outcome was graded as very low, due to serious risk of bias and imprecision.

Five studies comprising 16 225 patients reported on all-cause mortality up to 6 months after starting anticoagulation. There were 76 deaths in patients given a DOAC compared with 86 in those treated with a VKA (OR: 1.00, CI: 0.73–1.37, I^2^: 0%). Risk of bias was rated as high in four studies in this analysis and moderate in two. Level of evidence for this outcome was graded as low, due to very serious risk of bias.

### Admission duration

*Figure 2*, panel C illustrates the relative effect of DOAC vs. VKA therapy on admission duration. Four studies comprising 16 175 patients reported on admission duration. Mean or median admission durations ranged from 0 to 13.7 days with high heterogeneity (I^2^ = 66%). The pooled mean difference was −0.33 (CI −1.16–0.49). Risk of bias was rated as high in three studies in this analysis and moderate in one. Level of evidence for this outcome was graded as very low, due to very serious risk of bias and inconsistency and serious imprecision.

### Reintervention and readmission

Two studies on a total of 128 patients reported zero reinterventions in their population, with consequently no difference between both arms. Risk of bias was rated as high in one study in this analysis and moderate in one. Level of evidence for this outcome was graded as very low due to serious risk of bias and very serious imprecision.

One study reported readmission rates for our population of interest. This study comprised 1743 patients and evaluated readmission rates at 1-month follow-up. In the DOAC group, 89 out of 570 patients (15.6%) were readmitted compared with 207 out of 1173 (17.6%) patients treated with VKAs.

### Summary of findings and secondary outcomes

*Table 2* summarizes our primary findings while our supplementary results are detailed in Supplementary material online, *Appendix F*. No differences in thromboembolic events, major bleeding, stroke, and all-cause mortality were observed between DOACs and VKAs at 12 months post-operative.

**Table 2:** Summary of primary findings

Outcomes	No. of participants (studies) follow-up	Certainty of the evidence (GRADE)	Relative effect (95% CI)	Anticipated absolute effects	
				Risk with VKA	Risk difference with DOAC
Major bleeding^a^	17 982 (9 studies)^b^	⨁ ○ ○ ○ Very low^c,d^	**OR 0.97** (0.60 to 1.56)	23 per 1.000^b^	**1 fewer per 1.000** (9 fewer to 13 more)
Thromboembolic events^a^	17 910 (8 studies)^b^	⨁ ○ ○ ○ Very low^c,d^	**OR 0.96** (0.62 to 1.50)	7 per 1.000^b^	**0 fewer per 1.000** (2 fewer to 3 more)
All-cause mortality^a^	16 225 (5 studies)^b^	⨁ ⨁ ○ ○ Low^c^	**OR 1.00** (0.73 to 1.37)	10 per 1.000^b^	**0 fewer per 1.000** (3 fewer to 4 more)
Admission duration^a^	16 175 (4 studies)	⨁ ○ ○ ○ Very low^c,d,f^	—	The mean admission duration was **6.63** days	MD **0.33 days lower** (1.16 lower to 0.49 higher)
Reintervention^a^	128 (2 studies)^b^	⨁ ○ ○ ○ Very low^e,g^	**OR 1.29** (0.08 to 21.03)	0 per 1.000^b^	**0 fewer per 1.000** (0 fewer to 0 more)
Any stroke^a^	2055 (4 studies)^b^	⨁ ○ ○ ○ Very low^c,d^	**OR 1.44** (0.61 to 3.41)	8 per 1.000^b^	**1 more per 1.000** (4 fewer to 13 more)

CI, confidence interval; MD, mean difference; OR, odds ratio.

^a^Follow-up: 0–6 months.

^b^Patients that develop the event in 6 months.

^c^ ≥ 50% of the contributing weight was at serious/high risk of bias.

^d^The overall 95% CI overlapped both important harm as important benefit but was smaller than 2.5.

^e^The overall 95% CI overlapped important harm and important benefit and was wider than 2.5.

^f^An I^2^ of more than 60% indicated considerable heterogeneity.

^g^≥ 25% of the contributing weight was at serious/high risk of bias.

Furthermore, no significant differences were observed between the two groups in any of the secondary outcomes. All secondary outcomes were graded as very low quality due to imprecision and bias.

### Subgroup and sensitivity analyses

Meta-regression (see Supplementary material online, *Tables F2*  *and F3*) showed that study design and surgery type did not significantly influence any outcomes. However, for admission duration, POAF emerged as a significant moderator, indicating that POAF patients treated with DOACs had longer admission durations compared with VKAs (coefficient: 1.0169, 95% CI: 0.3143–1.7195, *P* = 0.0046), with the model explaining all variance (*R*^2^=100%, QM = 8.0487, *P* = 0.00). AF type did not explain heterogeneity in other outcomes.

Subgroup analyses for the thromboembolic events and major bleeding outcomes are provided in Supplementary material online, *Appendix F*, with no significant differences observed between DOACs and VKAs across the subgroups. Sensitivity analyses confirmed the robustness of our primary findings.

## Discussion

This systematic review and meta-analysis encompassing 11 studies and over 18 000 patients yielded two major findings. First, it highlights the absence of high-quality evidence comparing DOACs with VKAs in patients with AF following cardiac surgery. Second, although the certainty of evidence is low, the available data do not suggest meaningful differences in efficacy or safety between DOACs and VKAs in this setting. This stands in contrast to prevailing preferences in clinical practice, where VKAs are often preferred in the early post-operative period. Notably, patients with a specific indication for VKAs were excluded.

Across all primary endpoints, including thromboembolic events and major bleeding and mortality, event rates were similar between treatment groups. Subgroup analyses revealed no significant differences in safety and efficacy between VKAs and DOACs, irrespective of AF type or surgical procedure. While major bleeding exhibited moderate between-study heterogeneity, none of the pre-specified subgroups significantly explained this variability. Admission duration, however, exhibited substantial between-study heterogeneity, which was further explored through *post-hoc* exploratory meta-regression. This analysis identified POAF as a significant moderator. Specifically, in patients with POAF, admission durations were longer for those treated with DOACs compared with those on VKAs. However, the effect size was low, and this finding was based on a limited number of studies and should be interpreted with caution.

### Clinical implications

These findings contribute meaningfully to the ongoing debate on OAC selection after cardiac surgery. In the absence of specific guidance for the early post-operative period, many centres continue to prefer VKAs after cardiac surgery.^6–12^ However, this preference is evolving due to the ease of DOAC dosing and advances in DOAC reversal. While our analyses did not uncover differences in outcomes, pharmacological distinctions between the drugs may help guide treatment decisions. VKAs require frequent INR monitoring due to significant inter-patient variability in dosing. Additionally, their delayed onset of effect, due to the prolonged half-life of factor II, may delay optimal anticoagulation in early treatment stages.^41–43^ In contrast, DOACs provide a more predictable response, with fixed dosing and minimal monitoring.^44^

Reversal strategies also differ. VKAs are irreversible antagonists of circulating vitamin K-dependent clotting factors and expedited reversal requires vitamin K or clotting factor suppletion through co-administering four factor concentrate (4F-PCC).^45^ DOACs have shorter half-lives than VKAs, often allowing reversal through discontinuation and supportive care alone. For DOACs, 4F-PCC is also an option, yielding comparable results to its use in VKA reversal.^46^  ^,47^ In addition, specific antidotes are now available to reverse DOACs – idarucizumab for dabigatran and Andexanet Alfa for the factor Xa inhibitors – though they remain expensive, have limited availability, and high-quality outcome data on using these reversal agents before emergency interventions is still scarce.^48–51^

Although the use of DOACs after cardiac surgery is increasing, many clinicians continue to favour VKAs over DOACs post-operatively, often due to a presumed safety advantage.^6–12^ However, we found no evidence suggesting a difference between DOACs and VKAs in this scenario.

Beyond the choice between DOACs and VKAs, post-operative antithrombotic therapy must be individualised based on surgical context. Patients with mechanical valve prostheses require lifelong VKA therapy. In patients with AF undergoing bioprosthetic valve replacement, DOACs are generally preferred over VKAs after the initial three months, but the optimal anticoagulation strategy during the early post-operative period remains uncertain.^52^ In patients post-CABG, guidelines recommend discontinuing combined OAC and antiplatelet therapy after 12 months if long-term anticoagulation is indicated; however, management strategies during the immediate post-operative phase are less clearly defined.^53^ Therefore, our findings provide new insights but should be interpreted alongside the need for individualised post-operative care based on surgical factors, bleeding risk, valve status, and concomitant antiplatelet indications.

### Strengths and limitations

This is the most comprehensive meta-analysis to date evaluating anticoagulant strategies in the early post-operative phase after cardiac surgery. Strengths include a robust literature search, extensive subgroup and sensitivity analyses, and independent assessment by two reviewers. We also performed comprehensive bias and quality assessment, which further strengthen the reliability of our findings. Additionally, our study provides valuable insights into an underexplored clinical scenario, focusing on the use of DOACs vs. VKAs in patients with AF in the months following cardiac surgery. Previous meta-analyses on this subject considered patients with long histories of cardiac surgery or patients post-percutaneous interventions.^21,22^ In contrast, our analysis focuses on patients who recently underwent cardiac surgery – a period when the risks and benefits of anticoagulation are most pronounced and clinical guidance remains limited.

Despite these strengths, several limitations warrant consideration. Most included cohorts were non-randomized, which increases uncertainty around our pooled estimates. Clinician preferences and perceived risks profiles likely influenced anticoagulant choice, particularly in the retrospective multicenter or national cohorts (i.e. Moser 2023^35^, Nauffal 2021^36^, Skogseid^39^). For example, DOACs may have been preferentially prescribed to patients perceived at lower thrombotic or bleeding risk, or to those already receiving DOACs prior to surgery, while VKAs may have been favoured in high-risk patients or in centres with established protocols favouring VKAs in the post-operative period. These prescribing patterns may introduce confounding by indication and limit comparability between groups. We accounted for these biases through structured risk-of-bias assessment using ROBINS-I and incorporated these judgments into GRADE evaluations, downgrading the certainty of evidence accordingly.

Furthermore, the lack of high-quality RCTs limits the strength of the available evidence, and low event rates likely reduced statistical power. Notably, reintervention rates were strikingly low, with zero reinterventions reported in two studies, whereas the average 30-day reintervention rate for complications following CABG in the Netherlands is 4.9%.^54^

Heterogeneity in the study population is another important consideration. Our analysis included both POAF and pre-existing AF patients, two populations with differing levels of evidence supporting anticoagulation. While AF and POAF differ in their underlying risk profiles and anticoagulation histories, anticoagulation decisions in the early post-operative period are often driven more by surgical context than by AF chronicity. Combining both groups in our analysis reflects this clinical reality, where OAC is frequently prescribed to both populations by cardiac surgeons during the index admission, in line with current guidelines that suggest managing POAF according to the same principles as other AF presentations or general post-operative anticoagulation strategies.^13,15,18^ Although pooling adds complexity to the interpretation of results, our subgroup analyses and meta-regression did not reveal evidence of differential treatment effects by AF type for any endpoint except admission duration. However, these exploratory findings should be interpreted only as hypothesis generating, given the limited number of studies. Nonetheless, our rigorous and systematic approach provides a pragmatic, focused, and up-to-date synthesis of the available evidence on OAC use in patients with AF following major cardiac surgery.

### Future directions

Future research should prioritise conducting large, high-quality RCTs to provide more robust evidence on the safety and efficacy of VKAs and DOACs after cardiac surgery. The ongoing DANCE trial (NCT04284839) and PACES trial (NCT04045665) offer promise in this regard. Further research is also warranted into anticoagulant reversal following cardiac surgery, with an emphasis on clinical outcomes and associated costs of reversal.

## Conclusion

There is no high-quality evidence available on the effect of DOACs vs. VKAs in patients with AF following cardiac surgery, and clinical decision-making must rely on suboptimal data. Our rigorous systematic review and meta-analysis did not reveal evidence of a meaningful difference in efficacy or safety between DOACs and VKAs. While awaiting high-quality randomized data, our meta-analysis found no evidence to support routinely avoiding DOACs or switching to VKAs in patients with AF undergoing cardiac surgery.

## Lead author biography


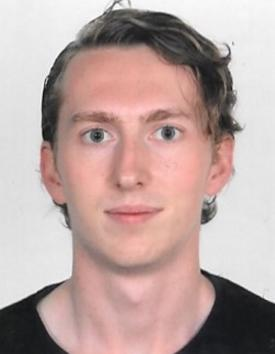


Marc Michiel Terpstra obtained his medical degree from the University of Amsterdam and is currently a research physician and PhD candidate in the Department of Cardiology at Amsterdam UMC. His research focuses on AF, with particular interest in stroke prevention and atrial structural remodelling.

## Supplementary Material

oeaf062_Supplementary_Data

## Data Availability

The data underlying this article will be shared on reasonable request to the corresponding author.
