# Supplementary material for: Does the type of oral anticoagulant matter for stroke prevention or bleeding in patients with atrial fibrillation after cardiac surgery? A systematic review and meta-analysis
Source: Eur Heart J Open. 2025 Jun 4;5(3):oeaf062. doi: 10.1093/ehjopen/oeaf062 (PMC12166522; doi:10.1093/ehjopen/oeaf062)
Supplement: oeaf062_Supplementary_Data [file oeaf062_supplementary_data.docx]

SUPPLEMENTARY APPENDIX

Table of Contents

[Supplementary Appendix A: PRISMA Flowchart and Checklist 2](#_Toc162432859)

[Supplementary Figure A1: 2](#_Toc162432859)

[Supplementary Table A1: 3](#_Toc162432860)

[Supplementary Appendix B: Search strategies 6](#_Toc162432861)

[Supplementary Table B1-B2: 6](#_Toc162432862)-7

[Supplementary Appendix C: Eligibility criteria 9](#_Toc162432864)

[Supplementary Appendix D: Considerations for bias and quality assessment 10](#_Toc162432867)

[Supplementary Appendix D.1 Considerations for Bias assessment 10](#_Toc162432868)

[Supplementary Table B1: 11](#_Toc162432869)

[Supplementary Appendix D.2 Considerations for quality assessment 12](#_Toc162432870)

[Supplementary Table B2: 12](#_Toc162432871)

[SUPPLEMENTARY APPENDIX E: Bias and quality assessment 14](#_Toc162432872)

[Supplementary Appendix E.1: Bias assessment results 14](#_Toc162432873)

[Supplementary Figure E1-E2:. 14](#_Toc162432874)

[Supplementary Appendix E.2: Quality assessment results 16](#_Toc162432876)

[Supplementary Table E1-E2: 16](#_Toc162432877)-16

[Supplementary Appendix F: Supplementary results 18](#_Toc162432879)

[Supplementary Appendix F.1 summary of secondary outcomes 18](#_Toc162432880)

[Supplementary Table F1: 18](#_Toc162432881)

[Supplementary Appendix F.2 secondary outcomes 18](#_Toc162432882)

[Supplementary Figure F1-F4. 18](#_Toc162432883)-19

[Supplementary Appendix F.3 Primary outcomes with 12 months follow up 20](#_Toc162432887)

[Supplementary Figure F5-F7. 20](#_Toc162432888)-21

[Supplementary Appendix F.4 Subgroup analyses 22](#_Toc162432891)

[Supplementary Figure F8-F20: 22](#_Toc162432892)-28

Supplementary Appendix F.5 Meta regression………………………………………..…………………………………….….. 29

Supplementary Table F2-F3………………………………………….…………………………………………………………….29-30

Supplementary Appendix F.6 Funnel plots…………………………………………………………..……………………………..31

Supplementary Figure F21-F29………………………………………….……………………………………………………….31-32
[Supplementary References..………………………………………………………………………………………………………..…………32](#_Toc162432904)

# Supplementary Appendix A: PRISMA Flowchart and Checklist


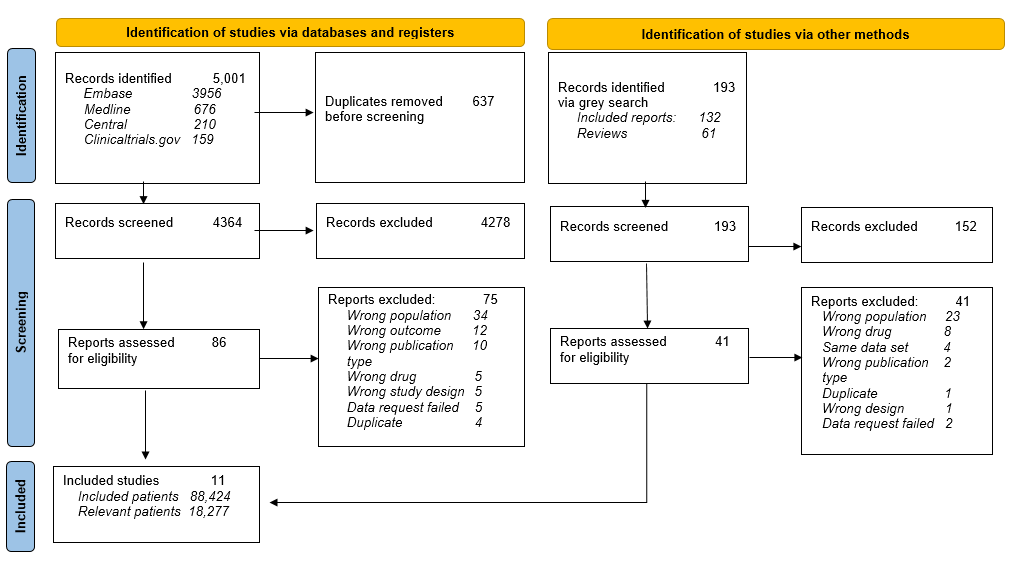


Supplementary Figure **A1:** PRISMA flowchart. Note that some records have more than one reason for exclusion, the most appropriate reason was depicted.

### Supplementary Table A1: PRISMA checklist

| **Section and Topic** | **Item #** | **Checklist item** | **Location where item is reported (page/Supplementary Appendix)** | |
| --- | --- | --- | --- | --- |
| **TITLE** | | |  | |
| Title | 1 | Identify the report as a systematic review. | 1 | |
| **ABSTRACT** | | |  | |
| Abstract | 2 | See the PRISMA 2020 for Abstracts checklist. | 2 | |
| **INTRODUCTION** | | |  | |
| Rationale | 3 | Describe the rationale for the review in the context of existing knowledge. | 3, 4 | |
| Objectives | 4 | Provide an explicit statement of the objective(s) or question(s) the review addresses. | 4 | |
| **METHODS** | | |  | |
| Eligibility criteria | 5 | Specify the inclusion and exclusion criteria for the review and how studies were grouped for the syntheses. | 5, 6, Supplementary Appendix C | |
| Information sources | 6 | Specify all databases, registers, websites, organisations, reference lists and other sources searched or consulted to identify studies. Specify the date when each source was last searched or consulted. | 5, Supplementary Appendix B | |
| Search strategy | 7 | Present the full search strategies for all databases, registers and websites, including any filters and limits used. | Supplementary Appendix B | |
| Selection process | 8 | Specify the methods used to decide whether a study met the inclusion criteria of the review, including how many reviewers screened each record and each report retrieved, whether they worked independently, and if applicable, details of automation tools used in the process. | 5 | |
| Data collection process | 9 | Specify the methods used to collect data from reports, including how many reviewers collected data from each report, whether they worked independently, any processes for obtaining or confirming data from study investigators, and if applicable, details of automation tools used in the process. | 6 | |
| Data items | 10a | List and define all outcomes for which data were sought. Specify whether all results that were compatible with each outcome domain in each study were sought (e.g. for all measures, time points, analyses), and if not, the methods used to decide which results to collect. | 5 | |
|  | 10b | List and define all other variables for which data were sought (e.g. participant and intervention characteristics, funding sources). Describe any assumptions made about any missing or unclear information. | 6 | |
| Study risk of bias assessment | 11 | Specify the methods used to assess risk of bias in the included studies, including details of the tool(s) used, how many reviewers assessed each study and whether they worked independently, and if applicable, details of automation tools used in the process. | 6, Supplementary Appendix D | |
| Effect measures | 12 | Specify for each outcome the effect measure(s) (e.g. risk ratio, mean difference) used in the synthesis or presentation of results. | 7, 8 | |
| Synthesis methods | 13a | Describe the processes used to decide which studies were eligible for each synthesis (e.g. tabulating the study intervention characteristics and comparing against the planned groups for each synthesis (item #5)). | 6,7 | |
|  | 13b | Describe any methods required to prepare the data for presentation or synthesis, such as handling of missing summary statistics, or data conversions. | 6. 7 | |
|  | 13c | Describe any methods used to tabulate or visually display results of individual studies and syntheses. | 7 | |
|  | 13d | Describe any methods used to synthesize results and provide a rationale for the choice(s). If meta-analysis was performed, describe the model(s), method(s) to identify the presence and extent of statistical heterogeneity, and software package(s) used. | 6, 7 | |
|  | 13e | Describe any methods used to explore possible causes of heterogeneity among study results (e.g. subgroup analysis, meta-regression). | 6-8 | |
|  | 13f | Describe any sensitivity analyses conducted to assess robustness of the synthesized results. | 7, 8, Table 1 | |
| Reporting bias assessment | 14 | Describe any methods used to assess risk of bias due to missing results in a synthesis (arising from reporting biases). | 7, Supplementary Appendix D | |
| Certainty assessment | 15 | Describe any methods used to assess certainty (or confidence) in the body of evidence for an outcome. | 6-8 Supplementary Appendix D | |
| **RESULTS** | | |  | |
| Study selection | 16a | Describe the results of the search and selection process, from the number of records identified in the search to the number of studies included in the review, ideally using a flow diagram. | 8, Supplementary Appendix A | |
|  | 16b | Cite studies that might appear to meet the inclusion criteria, but which were excluded, and explain why they were excluded. | Supplementary Appendix A | |
| Study characteristics | 17 | Cite each included study and present its characteristics. | 8, Table 1, Supplementary Appendix E | |
| Risk of bias in studies | 18 | Present assessments of risk of bias for each included study. | Supplementary Appendix E | |
| Results of individual studies | 19 | For all outcomes, present, for each study: (a) summary statistics for each group (where appropriate) and (b) an effect estimate and its precision (e.g. confidence/credible interval), ideally using structured tables or plots. | 8-11, Figures 1-2, Table 2, Supplementary Appendix F | |
| Results of syntheses | 20a | For each synthesis, briefly summarise the characteristics and risk of bias among contributing studies. | 8-11, Table 2, Supplementary Appendix F | |
|  | 20b | Present results of all statistical syntheses conducted. If meta-analysis was done, present for each the summary estimate and its precision (e.g. confidence/credible interval) and measures of statistical heterogeneity. If comparing groups, describe the direction of the effect. | 8-11, Figures 1-2, Table 2, Supplementary Appendix F | |
|  | 20c | Present results of all investigations of possible causes of heterogeneity among study results. | 11, Supplementary Appendix E, F | |
|  | 20d | Present results of all sensitivity analyses conducted to assess the robustness of the synthesized results. | 11, Supplementary Appendix E, F | |
| Reporting biases | 21 | Present assessments of risk of bias due to missing results (arising from reporting biases) for each synthesis assessed. | Supplementary Appendix E, F | |
| Certainty of evidence | 22 | Present assessments of certainty (or confidence) in the body of evidence for each outcome assessed. | Supplementary Appendix E | |
| **DISCUSSION** | | | |  |
| Discussion | 23a | Provide a general interpretation of the results in the context of other evidence. | | 11-15 |
|  | 23b | Discuss any limitations of the evidence included in the review. | | 14, 15 |
|  | 23c | Discuss any limitations of the review processes used. | | 14,15 |
|  | 23d | Discuss implications of the results for practice, policy, and future research. | | 11-15 |
| **OTHER INFORMATION** | | | |  |
| Registration and protocol | 24a | Provide registration information for the review, including register name and registration number, or state that the review was not registered. | | 2, 5 |
|  | 24b | Indicate where the review protocol can be accessed, or state that a protocol was not prepared. | | 5 |
|  | 24c | Describe and explain any amendments to information provided at registration or in the protocol. | | Table 1 |
| Support | 25 | Describe sources of financial or non-financial support for the review, and the role of the funders or sponsors in the review. | | 16 |
| Competing interests | 26 | Declare any competing interests of review authors. | | 16 |
| Availability of data, code and other materials | 27 | Report which of the following are publicly available and where they can be found: template data collection forms; data extracted from included studies; data used for all analyses; analytic code; any other materials used in the review. | | 16 |

# Supplementary Appendix B: Search strategies

### **Supplementary Table B1:** MEDLINE search strategy

| Database(s): Ovid MEDLINE(R) ALL 1946 to June 19, 2023, Update June 26, 2024 | |  |
| --- | --- | --- |
| Search Strategy: | |  |
| # | Searches | Results |
| 1 | exp atrial fibrillation/ or exp atrial flutter/ | 73865 |
| 2 | ((atri* or auricular) adj2 (fibril* or flutter*)).ti,ab,kf. | 96838 |
| 3 | (AF or "a fib" or afib or AFL).ti,ab,kf. | 54638 |
| 4 | or/1-3 | 126772 |
| 5 | exp Cardiovascular Surgical Procedures/ or exp Thoracic Surgical Procedures/ or exp Heart Valve Prosthesis/ | 599561 |
| 6 | ((cardiac or heart or thorax or cardiovascular* or cardiothoracic or myocardial or thoracic or chest) adj3 (surger* or surgical or operat* or repair* or correct* or resect* or procedure*)).ti,ab,kf. | 151422 |
| 7 | ((bioprosthetic or biological or tissue) adj3 (mitral or bicuspid) adj2 valve* adj3 (surger* or surgical or operat* or repair* or correct* or resect* or procedure*)).ti,ab,kf. | 52 |
| 8 | (BMV or BMVR or BAV or BAVR or BTV or BTVR or BPHV or BPHVR).ti,ab,kf. | 4968 |
| 9 | (maze adj2 (surger* or surgical or operat* or repair* or correct* or resect* or procedure*)).ti,ab,kf. | 1615 |
| 10 | ((LAA or LAAC or LLAO or "left atri*") adj2 (closure or surger* or surgical or operat* or repair* or correct* or resect* or procedure* or occlusion*)).ti,ab,kf. | 3582 |
| 11 | or/5-10 | 681563 |
| 12 | 4 and 11 | 16390 |
| 13 | *anticoagulants/ or exp antithrombins/ or exp factor xa inhibitors/ | 70101 |
| 14 | (NOAC* or DOAC* or antithrombo* or anticoagulant or ((anti or inhibit*) adj2 (coagulan* or thromb*))).ti,ab,kf. | 91146 |
| 15 | (factor adj2 ("xa inhibitor*" or "10a inhibitor*")).ti,ab,kf. | 2865 |
| 16 | (antistasin or apixaban or betrixaban or darexaban or edoxaban or eribaxaban or fidexaban or letaxaban or heparin or otamixaban or razaxaban or rivaroxaban or tanogitran or yagin or xarelto or dabigatran or pradaxa or apixaban or eliquis or lixiana or savaysa).ti,ab,kf. | 96696 |
| 17 | (Argatroban or Atecegatran or Bivalirudin or Bothrojaracin or Dabigatran or Dabigatran Etexilate or Desulfatohirudin or Dysinosin A or Efegatran or Flovagatran or Hirudin or Hirugen or Inogatran or Lepirudin or Melagatran or Napsagatran or Odiparcil or Pegmusirudin or Sofegatran or Tanogitran or Ximelagatran).ti,ab,kf. | 12343 |
| 18 | or/13-17 | 193868 |
| 19 | exp Coumarins/ | 56372 |
| 20 | ("vitamin k" adj2 (antagonist* or anti or antitromb*)).ti,ab,kf. | 8240 |
| 21 | (befarin or carfin or circuvit or c?umadan or c?umadin* or coumafene or coumaphene or digonal or farin or kumatox or maforan or marevan or orfarin or panwarfarin or panwarfin or prothromadin or neo sintrom or neosintrom or neositron or nic?umalon* or niffc?umar or nitrovarfarian or nitrowarfarin or brodifacoum or bromadiolone or cloricromen or c?umafos or c?umatetralyl or c?umetarol or dic?umarol or difenacoum or floc?umafen or phenproc?umon or phepromaron or tioclomarol or warfarin* or acenocoumarol or nicoumalone or acenocoumarin or sinthrome or sync?umar or sink?umar or sintrom or marc?umar or fluindione or liquamar or warfarin or aldocumar or coumadin* or jantoven or hydroxycoumarin* or coumarin* or acenocoumarol or acitrom or "antivitamins K").ti,ab,kf. | 49533 |
| 22 | or/19-21 | 83651 |
| 23 | 12 and 18 and 22 | 941 |
| 24 | exp controlled clinical trial/ or cross-over studies/ or double-blind method/ or random allocation/ or single-blind method/ or exp Cohort Studies/ or exp Clinical Trials as Topic/ or exp Equivalence Trial/ or exp Randomized Controlled Trials as Topic/ | 3424646 |
| 25 | ((singl* or doubl* or tripl*) adj2 (blind* or dumm* or mask*)).ti,ab,kf. | 198965 |
| 26 | ((control* or multicenter or crossover or "cross over" or clinical* or "intention to treat" or equivalence) adj2 (study or studies or trial* or group*)).ti,ab,kf. | 1833288 |
| 27 | (randomized or randomly or sham or placebo* or allocat*).ti,ab,kf. | 1274281 |
| 28 | drug therapy.fs. | 2601228 |
| 29 | (phase adj1 ("II" or "III" or "IV" or "2" or "3" or "4")).ti,ab,kf. | 142476 |
| 30 | ((clinical or pragmatic) adj2 trial*).ti,ab,kf. | 498851 |
| 31 | ((cohort* or longitudinal* or follow up of followup or prospective or retrospective) adj2 (stud* or design* or analy* or survey*)).ti,ab,kf. | 1216756 |
| 32 | or/24-31 | 6948933 |
| 33 | 23 and 32 | 741 |
| 34 | (Percutaneous or transcutaneous or ablation).ti. | 129902 |
| 35 | 33 not 34 | 604 |
|  |  |  |
| 36 | Update 20-6-2023 to 26-06-2024 | 72 |

### Supplementary Table B2: Embase search strategy

| Database(s): Embase Classic+Embase 1947 to June 19 2023 | |  |
| --- | --- | --- |
| Search Strategy: | |  |
| # | Searches | Results |
| 1 | exp atrial fibrillation/ or exp heart atrium flutter/ | 138681 |
| 2 | ((atri* or auricular) adj2 (fibril* or flutter*)).ti,ab,kf. | 178895 |
| 3 | (AF or "a fib" or afib or AFL).ti,ab,kf. | 106698 |
| 4 | or/1-3 | 246767 |
| 5 | exp cardiovascular surgery/ or exp thorax surgery/ or exp mitral valve prosthesis/ or exp mitral valve replacement/ or exp cardiovascular procedure/ | 1329335 |
| 6 | ((cardiac or heart or thorax or cardiovascular* or cardiothoracic or myocardial or thoracic or chest) adj3 (surger* or surgical or operat* or repair* or correct* or resect* or procedure*)).ti,ab,kf. | 231001 |
| 7 | ((bioprosthetic or biological or tissue) adj3 (mitral or bicuspid) adj2 valve* adj3 (surger* or surgical or operat* or repair* or correct* or resect* or procedure*)).ti,ab,kf. | 95 |
| 8 | (BMV or BMVR or BAV or BAVR or BTV or BTVR or BPHV or BPHVR).ti,ab,kf. | 7374 |
| 9 | (maze adj2 (surger* or surgical or operat* or repair* or correct* or resect* or procedure*)).ti,ab,kf. | 2403 |
| 10 | ((LAA or LAAC or LLAO or "left atri*") adj2 (closure or surger* or surgical or operat* or repair* or correct* or resect* or procedure* or occlusion*)).ti,ab,kf. | 6586 |
| 11 | or/5-10 | 1406190 |
| 12 | 4 and 11 | 76734 |
| 13 | exp blood clotting factor 10a inhibitor/ or exp thrombin inhibitor/ or *anticoagulant agent/ | 181239 |
| 14 | (NOAC* or DOAC* or antithrombo* or anticoagulant or ((anti or inhibit*) adj2 (coagulan* or thromb*))).ti,ab,kf. | 147871 |
| # | Searches | Results |
| 15 | (factor adj2 ("xa inhibitor*" or "10a inhibitor*")).ti,ab,kf. | 4731 |
| 16 | (antistasin or apixaban or betrixaban or darexaban or edoxaban or eribaxaban or fidexaban or letaxaban or heparin or otamixaban or razaxaban or rivaroxaban or tanogitran or yagin or xarelto or dabigatran or pradaxa or apixaban or eliquis or lixiana or savaysa).ti,ab,kf. | 149285 |
| 17 | (Argatroban or Atecegatran or Bivalirudin or Bothrojaracin or Dabigatran or Dabigatran Etexilate or Desulfatohirudin or Dysinosin A or Efegatran or Flovagatran or Hirudin or Hirugen or Inogatran or Lepirudin or Melagatran or Napsagatran or Odiparcil or Pegmusirudin or Sofegatran or Tanogitran or Ximelagatran).ti,ab,kf. | 21713 |
| 18 | or/13-17 | 346366 |
| 19 | antivitamin K/ or exp coumarin anticoagulant/ | 134763 |
| 20 | ("vitamin k" adj2 (antagonist* or anti or antitromb*)).ti,ab,kf. | 14498 |
| 21 | (befarin or carfin or circuvit or c?umadan or c?umadin* or coumafene or coumaphene or digonal or farin or kumatox or maforan or marevan or orfarin or panwarfarin or panwarfin or prothromadin or neo sintrom or neosintrom or neositron or nic?umalon* or niffc?umar or nitrovarfarian or nitrowarfarin or brodifacoum or bromadiolone or cloricromen or c?umafos or c?umatetralyl or c?umetarol or dic?umarol or difenacoum or floc?umafen or phenproc?umon or phepromaron or tioclomarol or warfarin* or acenocoumarol or nicoumalone or acenocoumarin or sinthrome or sync?umar or sink?umar or sintrom or marc?umar or fluindione or liquamar or warfarin or aldocumar or coumadin* or jantoven or hydroxycoumarin* or coumarin* or acenocoumarol or acitrom or "antivitamins K").ti,ab,kf. | 78334 |
| 22 | or/19-21 | 163772 |
| 23 | 12 and 18 and 22 | 5670 |
| 24 | exp controlled clinical trial/ or exp crossover procedure/ or exp double blind procedure/ or exp intention to treat analysis/ or exp single blind procedure/ or exp triple blind procedure/ or exp randomization/ or exp equivalence trial/ or exp clinical trial/ or exp "clinical trial (topic)"/ or exp cohort analysis/ or longitudinal study/ or prospective study/ or retrospective study/ or exp follow up/ | 6167616 |
| 25 | ((control* or multicenter or crossover or "cross over" or clinical* or "intention to treat" or equivalence) adj2 (study or studies or trial* or group*)).ti,ab,kf. | 2691025 |
| 26 | ((singl* or doubl* or tripl*) adj2 (blind* or dumm* or mask*)).ti,ab,kf. | 291218 |
| 27 | (randomized or randomly or sham or placebo* or allocat*).ti,ab,kf. | 1821701 |
| 28 | drug therapy.fs. | 4544668 |
| 29 | (phase adj1 ("II" or "III" or "IV" or "2" or "3" or "4")).ti,ab,kf. | 280472 |
| 30 | ((clinical or pragmatic) adj2 trial*).ti,ab,kf. | 747939 |
| 31 | ((cohort* or longitudinal* or follow up of followup or prospective or retrospective) adj2 (stud* or design* or analy* or survey* or trial*)).ti,ab,kf. | 1949892 |
| 32 | or/24-31 | 11172310 |
| 33 | 23 and 32 | 4730 |
| 34 | (Percutaneous or transcutaneous or ablation).ti. | 195771 |
| 35 | 33 not 34 | 3619 |
|  |  |  |
| 36 | Update search 20-6-2023 to 02-07-2024 | 337 |

# Supplementary Appendix C: Eligibility criteria

## Inclusion criteria:

- Studies reporting on cardiothoracic surgical procedures, through thoracotomy or thoracoscopy, comparing postoperative use of DOACs with VKAs, Specifically dabigatran and/or apixaban and/or rivaroxaban and/or edoxaban compared with warfarin and/or acenocoumarol and/or phenprocoumon.
- Studies reporting on atrial fibrillation or atrial flutter, either pre-existent or POAF.
- Studies reporting on events accrued in the 6 months postoperative period.
- Studies with the following publication types
  - Randomized controlled trials (RCT), cohort studies, case-control studies

## Exclusion criteria:

- Studies with non-human participants included.
- Studies reported solely in languages other than English or Dutch.
- Studies reporting on less than 50 patients meeting our criteria.
- Notes, editorials, cross-sectional studies, case-reports, phase 1 or 2 clinical trials, practice guidelines, systematic or scoping reviews, abstracts and design papers.
- Reports on included studies without additional relevant data
- Studies focussing on the following contraindications for study medications:
- Preoperative moderate to severe mitral stenosis
- Preoperative rheumatic mitral stenosis
- Mechanical heart valve
- Severe Kidney failure (estimated glomerular filtration rate (eGFR) <15 ml/min)
- Acute liver failure
- End-stage liver disease
- Endocarditis
- Sepsis
- Left ventricular assist device (LVAD)
- Pregnancy
- Studies reporting on percutaneous, ablation, and/or device-related procedures when not combined with cardiac surgery (e.g., surgical valve replacement).
- Studies focussing on patients with a competing indication for treatment with an OAC
- Treatment or prevention of venous thromboembolism
- Antiphospholipid syndrome

# Supplementary Appendix D: Considerations for bias and quality assessment

## Supplementary Appendix D.1 Considerations for Bias assessment

Bias assessments were performed at the domain level by two independent reviewers (MT and TdV) in duplicate. Following the domain level assessment, we assigned overall risks of bias regarding the study level.

Randomised studies were assessed for bias in the following domains: the randomisation process, deviations from intended interventions, missing outcome data, measurement of the outcome and selection of the reported result. The risk of bias for these domains was classified as low risk, some concerns or high risk. We used the RoB2-tool for bias ascertainment in these studies(1).

The non-randomised studies and studies that included a subgroup of a randomized study as population of interest were assessed for bias in these domains: confounding, selection of participants into the study, classification of interventions, deviations from intended interventions, missing data, measurement of outcomes and selection of reported data. The risk of bias for these domains was classified as low, moderate, high or critical risk or no information. With the exception of the confounding domain we used the ROBINS-I tool for bias ascertainment in these studies(2).

The preferred reporting of the effect was the assignment of effect using an intention to treat analysis. All studies were appraised from the perspective of adherence to an intention-to-treat analysis.

*Assessment of bias due to confounding for NRSI & subgroups of randomized studies:*

The robins-I guiding document recommends identifying potential confounding domains. Before starting our bias assessment we identified several domains that we deemed important confounders based on current literature or clinical rationale. We modified the SCRAP criteria and incorporated potential confounders of the relationship between the interventions of interest and thromboembolic and bleeding events. The domains are as follows:

- Sex
- Cardiovascular comorbidities affecting the risk of thromboembolisms
- Cardiovascular comorbidities affecting the risk of bleeding
- Comorbidities associated with pharmacokinetic properties that modify the anticoagulant effect of the studied drugs
- Race
- Age

We evaluated whether studies adequately addressed confounding by considering these domains. We differentiate between non randomized studies and subgroups of randomized studies.

*Non-randomized studies*

For non-randomized studies, we assigned at least a moderate bias for confounding if they were appropriately corrected for most of these domains. Otherwise, we considered at least a serious bias. If it was unclear how or for which domains the correction was performed, we assigned a serious label.

*Subgroups of randomized studies:*

Subgroups of randomized studies were evaluated as observational studies, using the ROBINS-I with the exception of the confounding domain. We speculated that studies had a low risk of bias due to confounding when (i) studies adequately randomized patients or clusters to the interventions of interest; and (ii) the potentially confounding characteristics were sufficiently similar between the subgroups of interest. Confounding was assed using the following pre-specified plan:

Supplementary Table D1: confounding assessment criteria for non-randomized studies and subgroups of randomized studies.

| **Low risk of bias** (the analysis of the subgroup is comparable to a well-performed  randomized trial with regard to this  domain) | (i) The study was randomized for the  interventions of interest (i.e. DOAC(s) vs VKA(s)).  *And*  (ii) Any baseline differences observed between  intervention groups in the subgroup appear to  be compatible with chance  We acknowledge that patients may still differ in non-measured confounders. However, when patients/clusters were randomized on the intervention of interest, we expect limited risk of residual confounding. |
| --- | --- |
| **Moderate risk of bias** (the analysis of the subgroup is sound for a non-randomized study  with regard to this domain but cannot be considered comparable to a well-  performed randomized trial) | (i.1) The study was randomized for the  interventions of interest.  *And*  (i.2) There is insufficient information about  baseline differences observed between  intervention groups in the subgroup*.*  *OR*  (ii.1) The study was not randomized for the  interventions of interest.  *And*  (ii.2) All pre-specified important confounding  Domains were appropriately measured and  Controlled for.  *And*  (ii.3) Reliability and validity of measurement of  important domains were sufficient, such that we  do not expect serious residual confounding. |
| **Serious risk of bias** (the study has some  important problems) | (i.1) The study was randomized for the  interventions of interest.  *And*  (i.2) Patients in two intervention groups differed  substantially in at least one pre-specified  confounding domain.  *Or*  (ii.1) The study was not randomized for the  interventions of interest.  *And*  *(ii.2.1) At least one* pre-specified important  confounding domain was not appropriately  measured and controlled for.  Or  *(ii.2.2)* Reliability or validity of measurement  of an important domain was insufficient so  that we expect serious residual confounding is  possible. |
| **Critical risk of bias** (the study is too  problematic to provide any useful  evidence on the effects of intervention) | (i.1) The study was not randomized for the  interventions of interest.  *And*  (ii.1) Confounding inherently not controllable.  Or  (ii.2) The use of negative controls strongly  suggests unmeasured confounding. |

## Supplementary Appendix D.2 Considerations for quality assessment

We formulated criteria for judgement of quality in adherence with the GRADE approach and based on the systematic review of de Vries et al(3, 4). Quality assessment was done at the outcome level. All studies included in our analyses started with high levels of evidence and were subsequently downgraded based on the criteria mentioned below, in line with GRADE guidelines 18.(5)

First, we evaluated study designs and potential sources of bias, and downgraded the evidence by one or two levels when there were serious or very serious limitations, respectively. Second, we assessed inconsistency using non-overlapping confidence intervals and I^2^ as indicators of heterogeneity. Third, we evaluated indirectness of the findings by assessing the applicability of each primary study to our predetermined research question. Next, we evaluated imprecision by assessing the confidence intervals of the pooled estimates, and whether the effects overlapped multiple directions of effect. Finally, we assessed publication bias using funnel plots. Funnel plots are presented in Supplementary Appendix F. Although we did not meet the commonly accepted cutoff of ten studies per outcome, we regarded funnel plots as the best available method to assess for potential publication bias. We did not upgrade levels of evidence.(4)

Supplementary Table B2: quality assessment criteria. Based on the GRADEpro handbook, GRADE guidelines 18, GRADE guidance 34, GRADE guidance 35, the Cochrane handbook for systematic reviews and the systematic review by de Vries et al.(3-8)

| **Domains** | **Considerations** | **Criteria for judgement** |
| --- | --- | --- |
| **Risk of bias** | The validity of our conclusion is most at risk when studies are flawed in their design or conduct. | Studies were assessed for their risk of bias using the criteria of either the Robins-I (observational studies, including subanalyses of randomized trials) or RoB2 tool (randomized controlled trials).  To rate quality of evidence for risk of bias across studies we considered the weight of the studies included in the analysis.  Per analysis, level of evidence was rated down by one grade if ≥25% of the contributing weight was at serious/high risk of bias.  Level of evidence was rated down by two grades if ≥25% of the contributing weight was from studies at critical risk or ≥50% from studies at serious/high risk of bias. |
| **Inconsistency** | Inconsistency refers to the presence of widely varying estimates or results across primary studies, unexplained by known factors or sources of variability. When there is substantial variation in point estimates and little to no overlap in confidence intervals, it is likely that certainty will be diminished due to inconsistency. | Per analysis we rated down the level of evidence if we found considerable heterogeneity among the point estimates of the included studies. We visually inspected the confidence intervals of the primary studies and evaluated the I^2^.  Level of evidence was rated down by one grade if there was either (a) limited overlap in the confidence intervals of the primary studies based on visual assessment or (b) if the I^2^ was ≥60%  Level of evidence was rated down by two grades if visual assessment of the confidence intervals pointed to important heterogeneity and I^2^ was ≥80%  When there was a discrepancy between the confidence intervals and the I^2^, the decision to rate down by one or two grades was determined my discussion among two reviewers (MT and TdV). |
| **Indirectness** | Indirectness refers to the extent to which the available evidence directly addresses the predetermined research question. A high level of indirectness limits our confidence and the applicability of the review's findings. | Indirectness was systematically evaluated in the following domains: comparisons, population, interventions and outcomes. We formulated questions in the PICO format for each study and compared these with our research question. Study indirectness was classified as: no indirectness, serious indirectness and very serious indirectness.  Level of evidence was rated down by one grade if ≥25% of contributing studies had serious indirectness.  Level of evidence was rated down by two grades if ≥25% of contributing studies had very serious indirectness or if ≥50% had serious indirectness. |
| **Imprecision** | Imprecision refers to a lack of accuracy in the estimated effect. When imprecision exists, this indicates that the true effect may be substantially different from the estimated effect. | We evaluated imprecision based om the minimally and partially contextualized approaches(6, 7). We established thresholds for serious harm and benefit in agreement. We assessed whether the confidence intervals of the effect sizes in our forest plots intersected no effect and these thresholds.  If a study outcome overlapped both no effect and clinically important harm or benefit, we rated down by one grade.  If the width of the confidence intervals of the odds ratio was more than 2.5, we rated down by one grade.  If outcomes fulfilled both criteria, we rated down by two grades.  Regarding continuous outcomes we rated down by two grades if the effect size overlapped both important harm and important benefit |
| **Publication Bias** | Selectively reporting results, either by not publishing entire reports or by selective publishing of analyses, can lower the certainty of evidence. | We plotted funnel plots to assess for potential publication bias. We acknowledge that this method is limited due to the small number of included studies.  We rated analyses as no serious risk or serious risk of publication bias through evaluation of the funnel plots.  Only if there was no alternative reasonable explanation for funnel plot asymmetry did we rate down the level of evidence by one grade. |

#

# SUPPLEMENTARY APPENDIX E: Bias and quality assessment

## Supplementary Appendix E.1: Funding and conflicts of interest

Supplementary Table E1: quality assessment criteria

| **Study** | **Funding** | **Conflicts of interest statement** |
| --- | --- | --- |
| Anderson et al, 2015 | Not reported. | Not reported. |
| Brochu et al, 2022 | The author(s) received no financial support for the research, authorship, and/or publication of this article. | The author(s) declared no potential conflicts of interest with respect to the research, authorship, and/or publication of this article. |
| Chapin et al, 2020 | The author(s) disclosed receipt of the following financial support for the research, authorship, and/or publication of this article: Funded by the Bristol-Myers Squibb and Pfizer Alliance (no role in study design or the collection, analysis, or interpretation of data). | The author(s) declared no potential conflicts of interest with respect to the research, authorship, and/or publication of this article. |
| Guimaraes et al, 2020 | Supported by the Brazilian Ministry of Health (PROADI-SUS) and Bayer. | Dr. Lopes reports receiving grant support, paid to his institution, and consulting fees from Bayer, Bristol Myers Squibb, GlaxoSmithKline, Medtronic, Pfizer, and Sanofi and consulting fees from Boehringer Ingelheim, Daiichi Sankyo, Merck, and Portola; Dr. de Barros e Silva, receiving grant support and lecture fees from Pfizer and Roche Diagnostics and grant support from Bayer; Dr. Alexander, receiving grant support, paid to his institution, and advisory board fees from Bayer and XaTek, grant support, paid to his institution, and consulting fees from Bristol Myers Squibb, grant support, paid to his institution, and honoraria from Cryolife, consulting fees from Janssen, consulting fees and honoraria from Pfizer, and fees for clinical event adjudication from Portola; and Dr. Berwanger, receiving grant support from AstraZeneca, Pfizer, Amgen, Servier, and Boehringer Ingelheim. No other potential conflict of interest relevant to this article was reported. |
| Moser et al, 2023 | No funding. | S.K. reported consulting fees from Pendopharm, HLS. W.K. reported Medtronic Canada consulting fees for valve surgery proctoring, Edwards consulting fees for a peer-to-peer training program, and Corcym also for a peer-to-peer training program. None of these conflicts are applicable to this manuscript. All other authors reported no conflicts of interest. |
| Nauffal et al, 2021 | Dr Lubitz is supported by NIH grant 1R01HL139731 and American Heart Association (AHA) grant 18SFRN34250007. This work was supported by the Fondation Leducq (14CVD01), NIH grants (1RO1HL092577, R01HL128914, K24HL105780), and AHA grant (18SFRN34110082) to Dr Ellinor. Dr Nauffal is supported by National Institutes of Health Training Grant in Cardiovascular Research (T32HL007604). | Dr Lubitz discloses a financial relationship with BMS/Pfizer, Boehringer Ingelheim, Bayer AG, Fitbit, and IBM; Dr Ellinor with Bayer AG, Novartis, MyoKardia and Quest. |
| Pasciolla et al, 2020 | Not reported. | Not reported. |
| Piepiorka-Broniecka et al, 2022 | Not reported. | None declared. |
| Shim et al, 2023 | The trial was funded by Daiichi Sankyo. | Dr De Caterina reports institutional grants from BMS/Pfizer and Daiichi-Sankyo; consultancy and speaker's fees from Boehringer-Ingelheim, Bayer, BMS/Pfizer, Daiichi Sankyo, Novartis, Portola, and Roche. All other authors reported no conflicts of interest. |
| Skogseid et al, 2024 | The authors have not declared a specific grant for this research from any funding agency in the public, commercial or not-for-profit sectors. | E-LS has no conflicts of interest. GB reports institutional research grants from Pfizer, expert committee and consulting fees to his institution from Bayer; honoraria for lectures and scientific advice from AstraZeneca, Boehringer Ingelheim, Bristol Myers Squibb, Novo Nordisk, Pfizer and Sanofi. JW has no conflicts of interest. CH reports institutional research grants from: Pfizer, GlaxoSmithKline, AstraZeneca and Bristol Myers Squibb; advisory board from: AstraZeneca, Bayer, Boehringer Ingelheim, Novo Nordisk and Coala Life; and personal fees from event adjudication (UCR). CC reports institutional research grant from Pfizer; speaker fees or advisory board from Bristol Myers Squibb, Bayer, Novartis, Orion Pharma and AstraZeneca; and personal fees from event adjudication (UCR). |
| Woldendorp et al, 2020 | No funding received. | There are no conflicts of interest declared for any authors on this manuscript. |

## Supplementary Appendix E.2: Bias assessment results


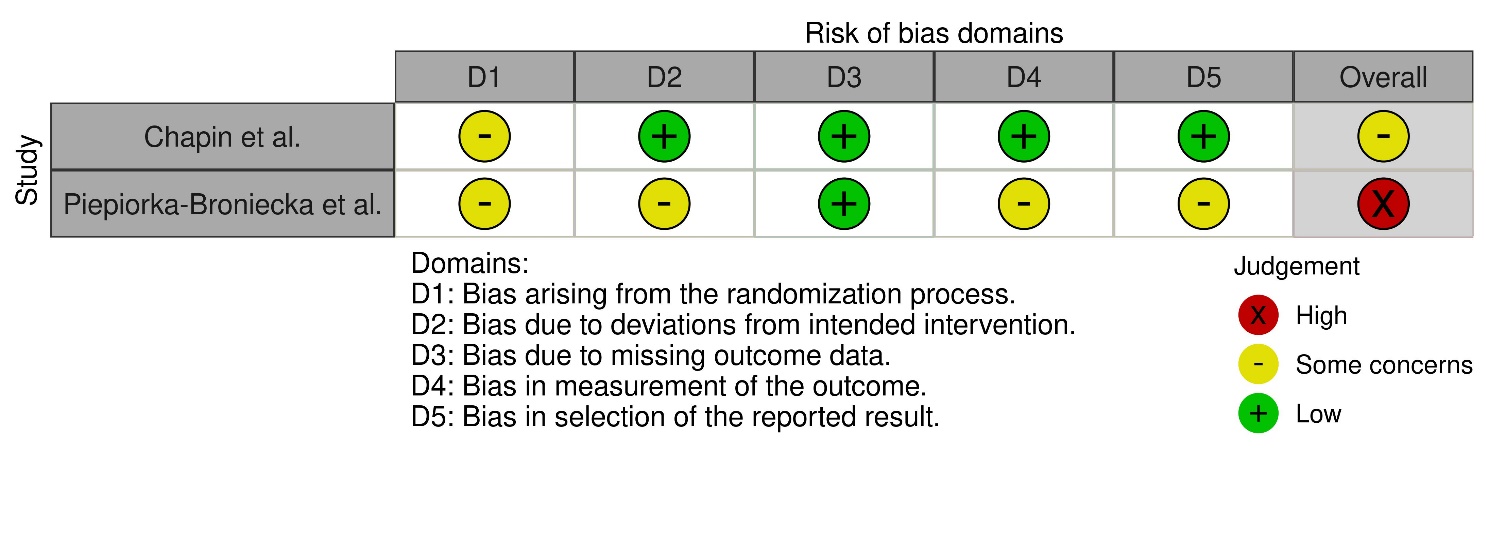


### Supplementary Figure E1: Bias assessment of randomized studies.

*
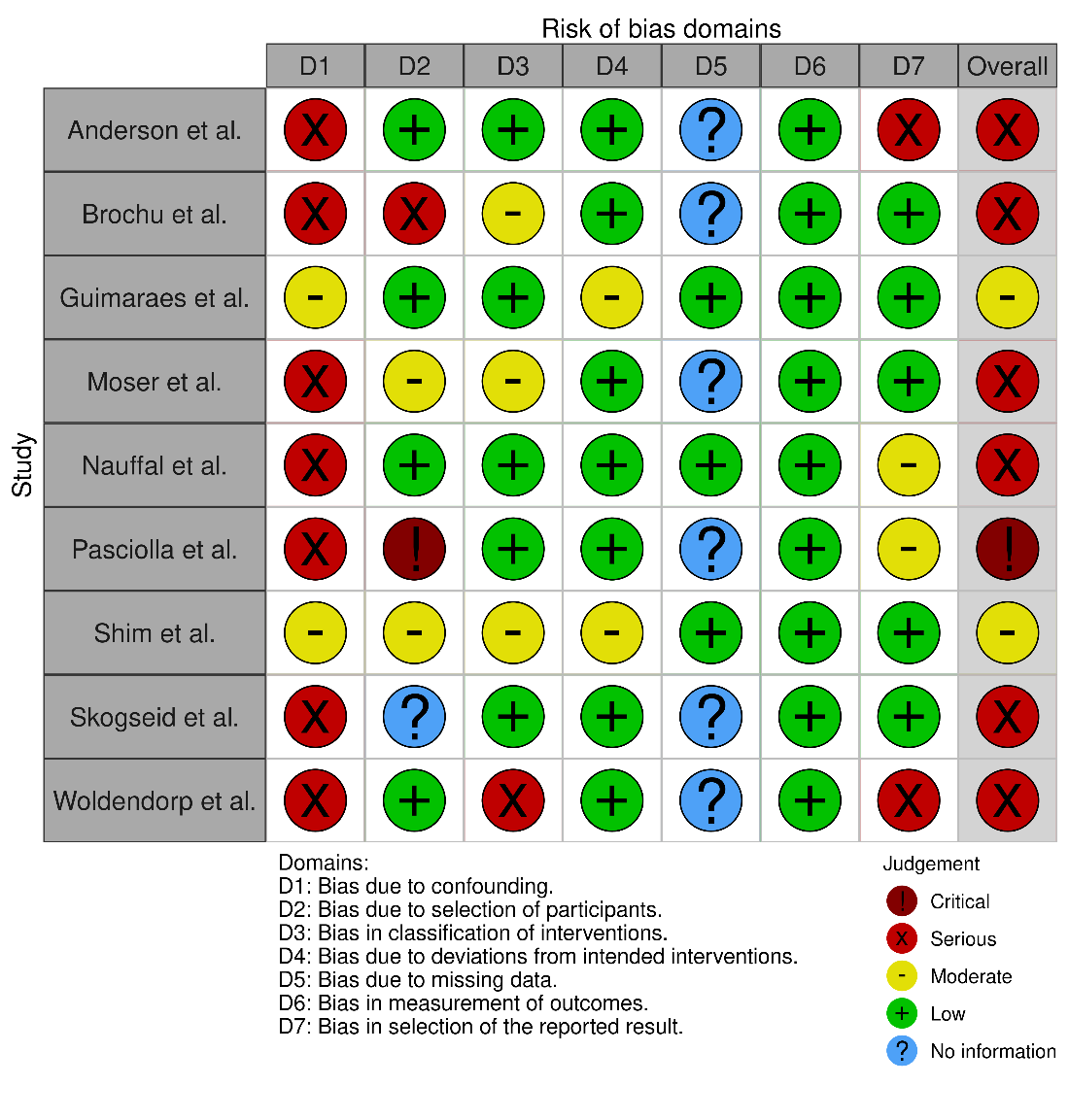
*

### Supplementary Figure E2: Bias assessment of non-randomized studies and subgroup analyses of randomized studies.

## Supplementary Appendix E.2: Quality assessment results

### Supplementary Table E2: GRADE quality assessment of primary outcomes

OR: Odds ratio, CI: Confidence interval. a: Follow-up: 0-6 months, b: Patients that develop the event in 6 months. c: Also including RCTs. d: ≥50% of the contributing weight was at serious/high risk of bias. e: The overall 95% CI overlapped both important harm as important benefit, but was smaller than 2.5. f: Risk differences could be calculated since no case-control studies were included. g: The overall 95% CI overlapped important harm and important benefit and was wider than 2.5. i: ≥25% of the contributing weight was at serious/high risk of bias.


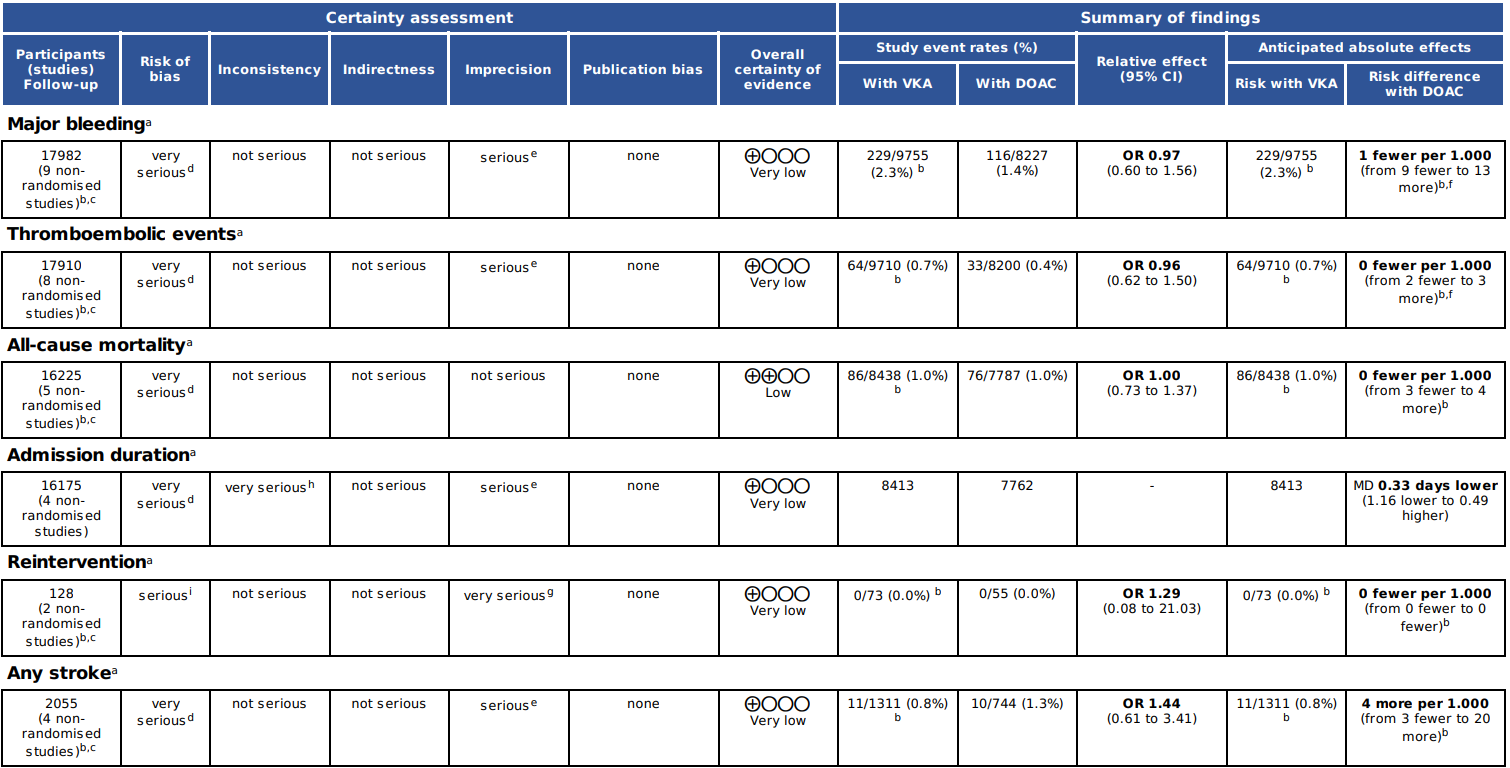


### Supplementary Table E3: GRADE quality assessment of secondary outcomes

OR: Odds ratio, CI: Confidence interval. a: Follow-up: 0-6 months, b: Patients that develop the event in 6 months. c: Also including RCTs. d: ≥50% of the contributing weight was at serious/high risk of bias. e: Risk differences could be calculated since no case-control studies were included. f: The overall 95% CI overlapped both important harm as important benefit, but was smaller than 2.5. g: ≥25% of the contributing weight was at serious/high risk of bias.


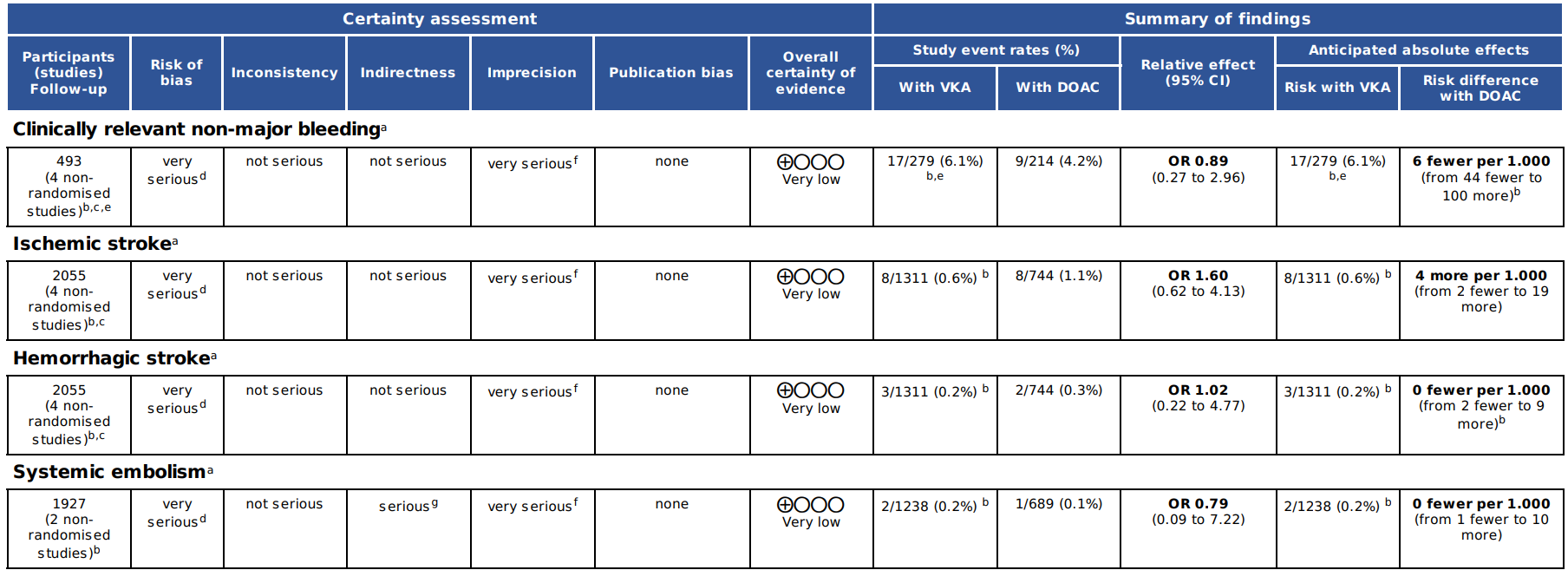


# Supplementary Appendix F: Supplementary results

## Supplementary Appendix F.1 summary of secondary outcomes

Supplementary Table F1: Summary of secondary findings. OR: Odds ratio, CI: Confidence interval. *: The risk in the intervention group (and its 95% confidence interval) is based on the assumed risk in the comparison group and the relative effect of the intervention (and its 95% CI). a: Follow-up: 0-6 months, b: Patients that develop the event in 6 months. c: Also including RCTs. d: ≥50% of the contributing weight was at serious/high risk of bias. e: The overall 95% CI overlapped both important harm as important benefit, but was smaller than 2.5. f: ≥25% of the contributing weight was at serious/high risk of bias*.*
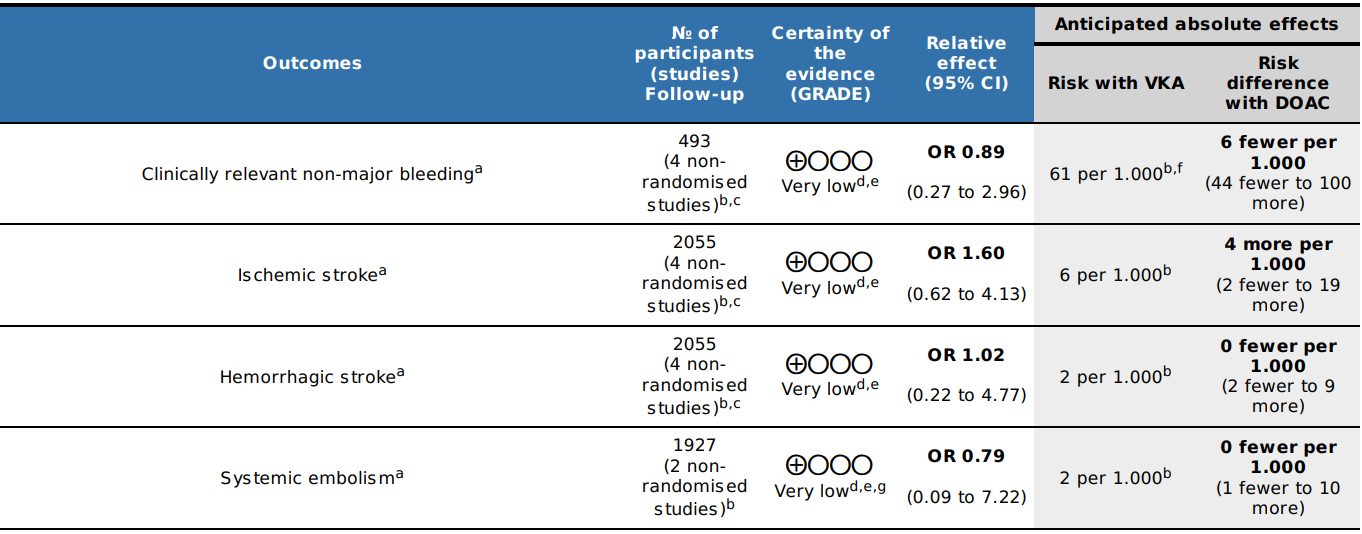


## Supplementary Appendix F.2 secondary outcome


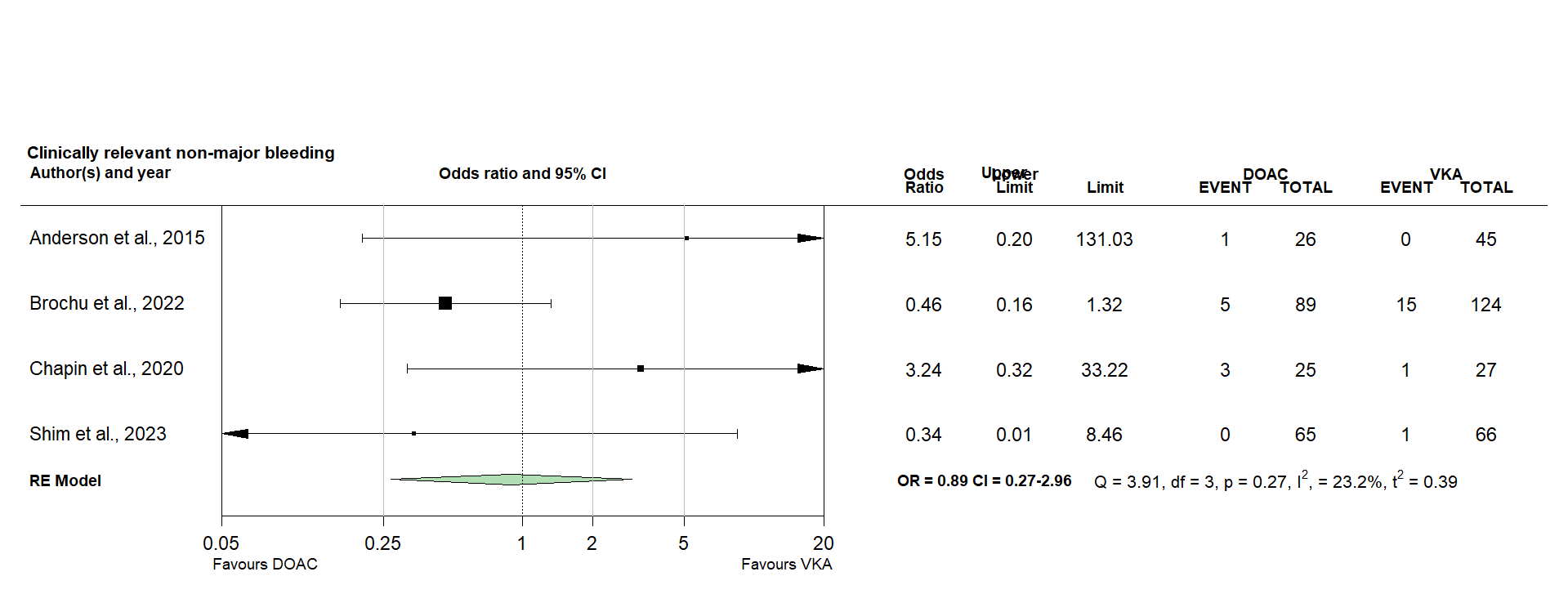


Supplementary Figure F1: Forest plot Clinically relevant non major bleeding up to 6 months after anticoagulation initiation. RE: Random effects. Q: Q statistic for effect size variability assessment. Df: Degrees of freedom. I^2^: Extent of Heterogeneity. t^2^: Between study variance estimate.


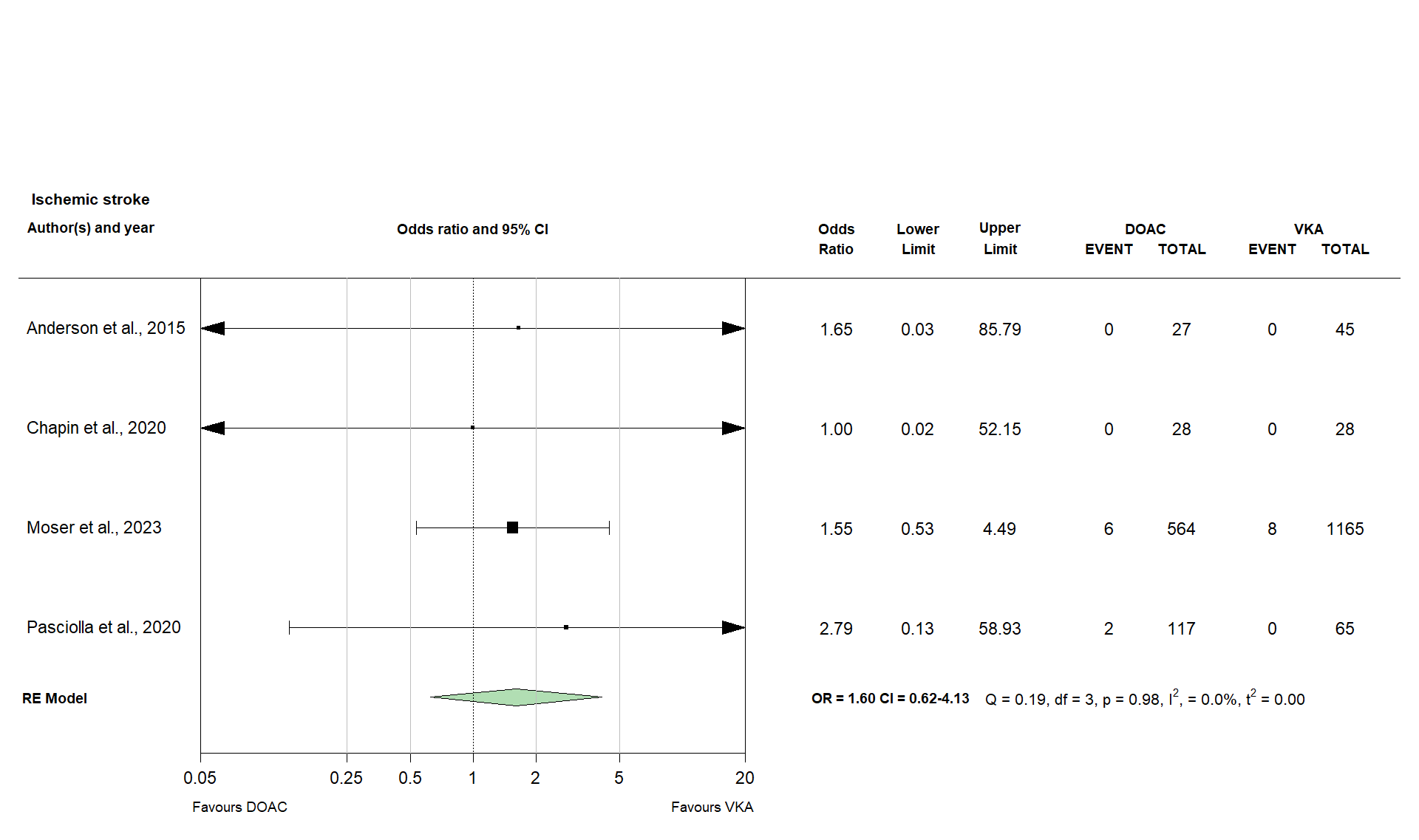
Supplementary Figure F2: Forest plot Ischemic stroke up to 6 months after anticoagulation initiation. RE: Random effects. Q: Q statistic for effect size variability assessment. Df: Degrees of freedom. I^2^: Extent of Heterogeneity. t^2^: Between study variance estimate.


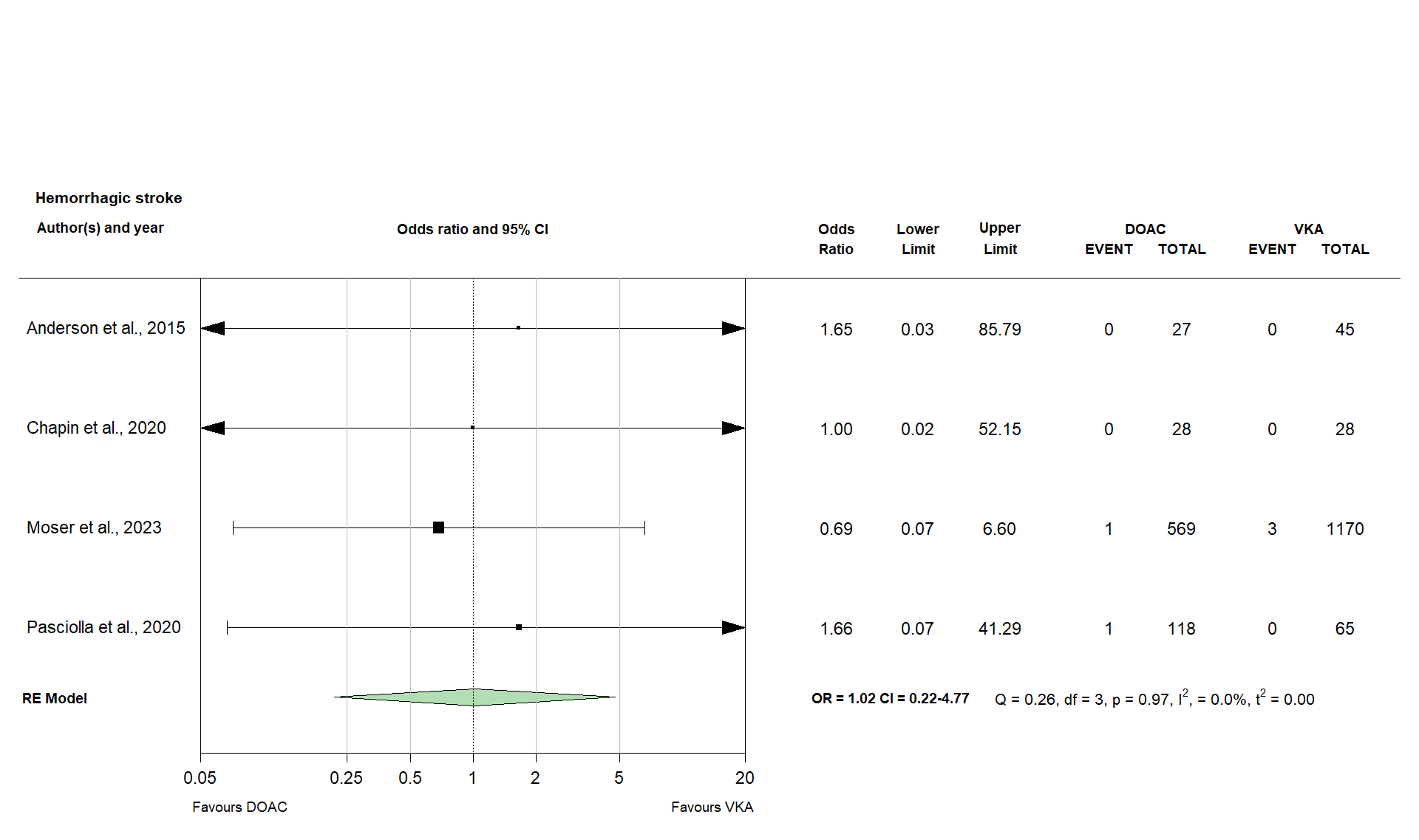
Supplementary Figure F3: Forest plot Hemorrhagic stroke up to 6 months after anticoagulation initiation. RE: Random effects. Q: Q statistic for effect size variability assessment. Df: Degrees of freedom. I^2^: Extent of Heterogeneity. t^2^: Between study variance estimate.


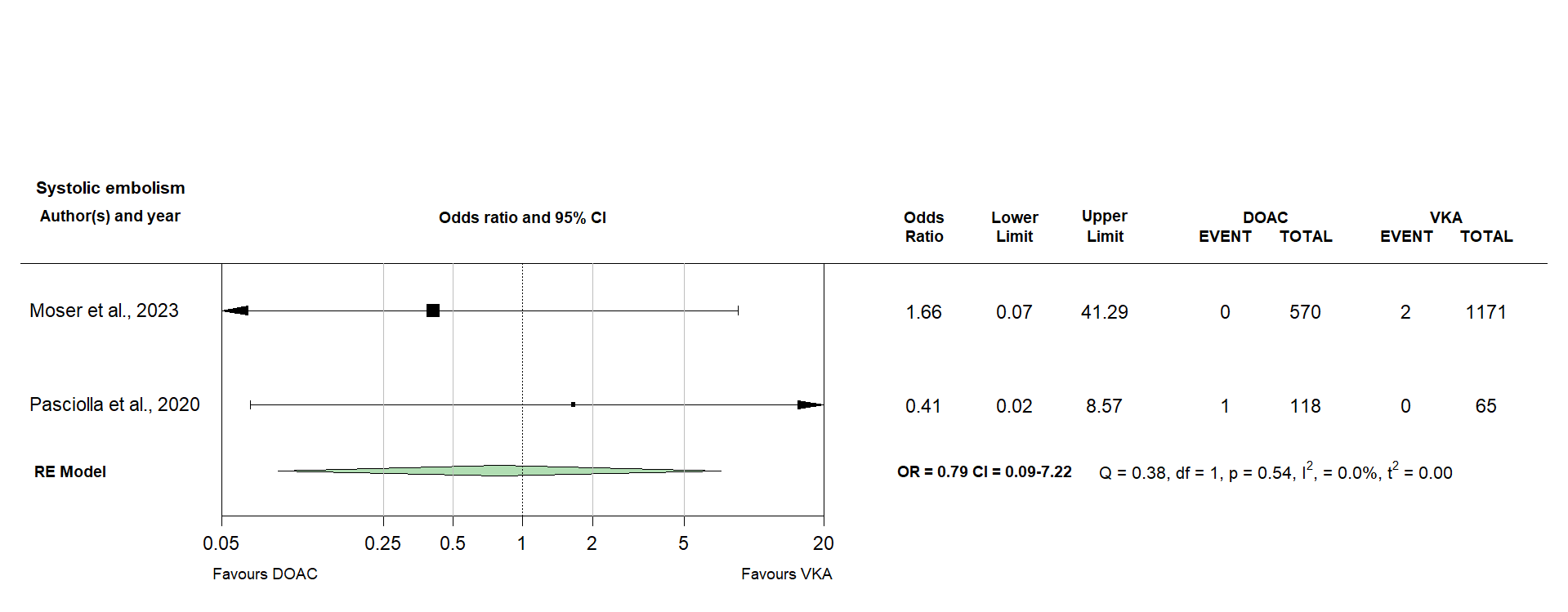


Supplementary Figure F4: Forest plot Systemic embolism up to 6 months after anticoagulation initiation. RE: Random effects. Q: Q statistic for effect size variability assessment. Df: Degrees of freedom. I^2^: Extent of Heterogeneity. t^2^: Between study variance estimate.


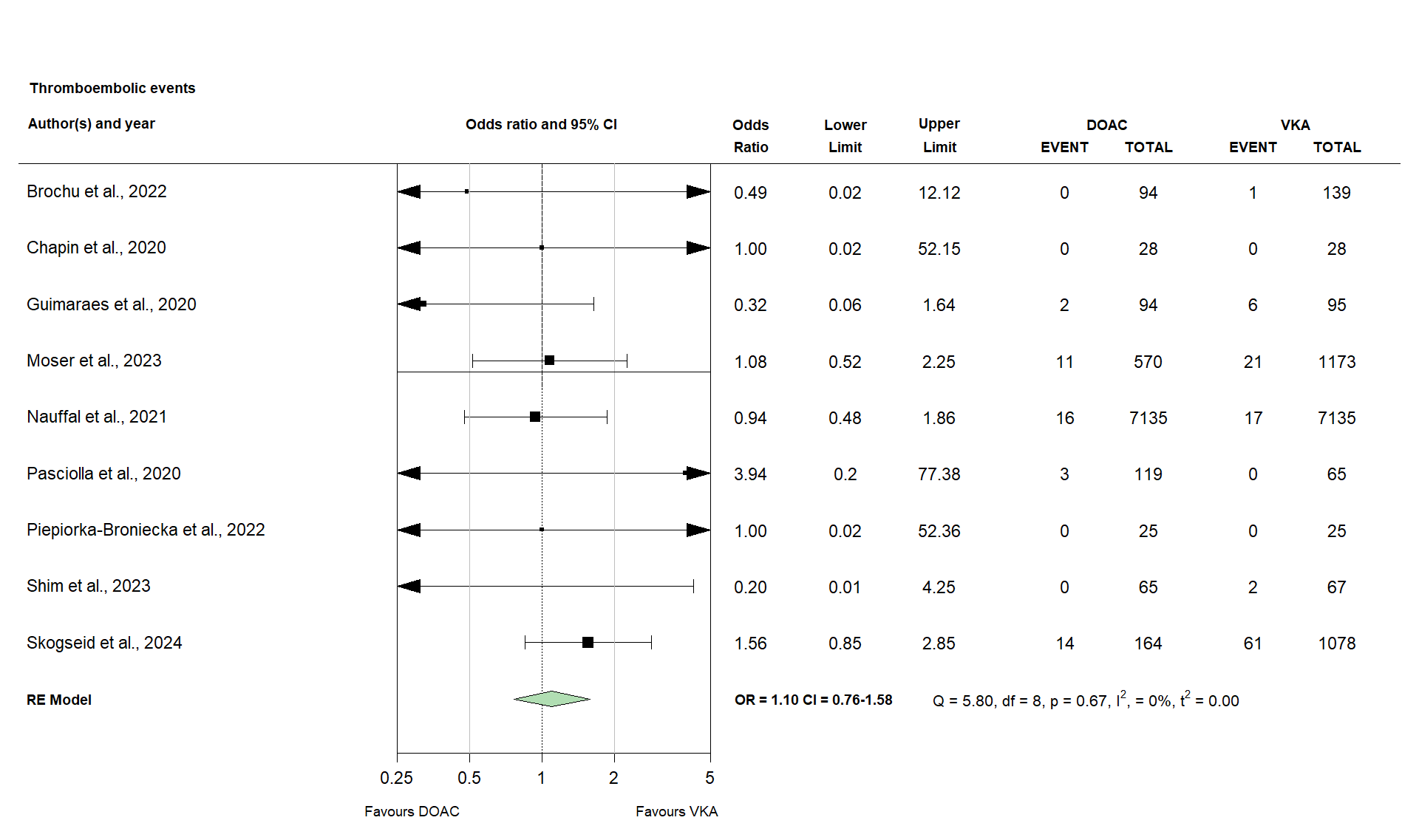


## Supplementary Appendix F.3 Primary outcomes with 12 months follow up

Supplementary Figure F5: Forest plot Thromboembolic events up to 12 months after anticoagulation initiation. RE: Random effects. Q: Q statistic for effect size variability assessment. Df: Degrees of freedom. I^2^: Extent of Heterogeneity. t^2^: Between study variance estimate.


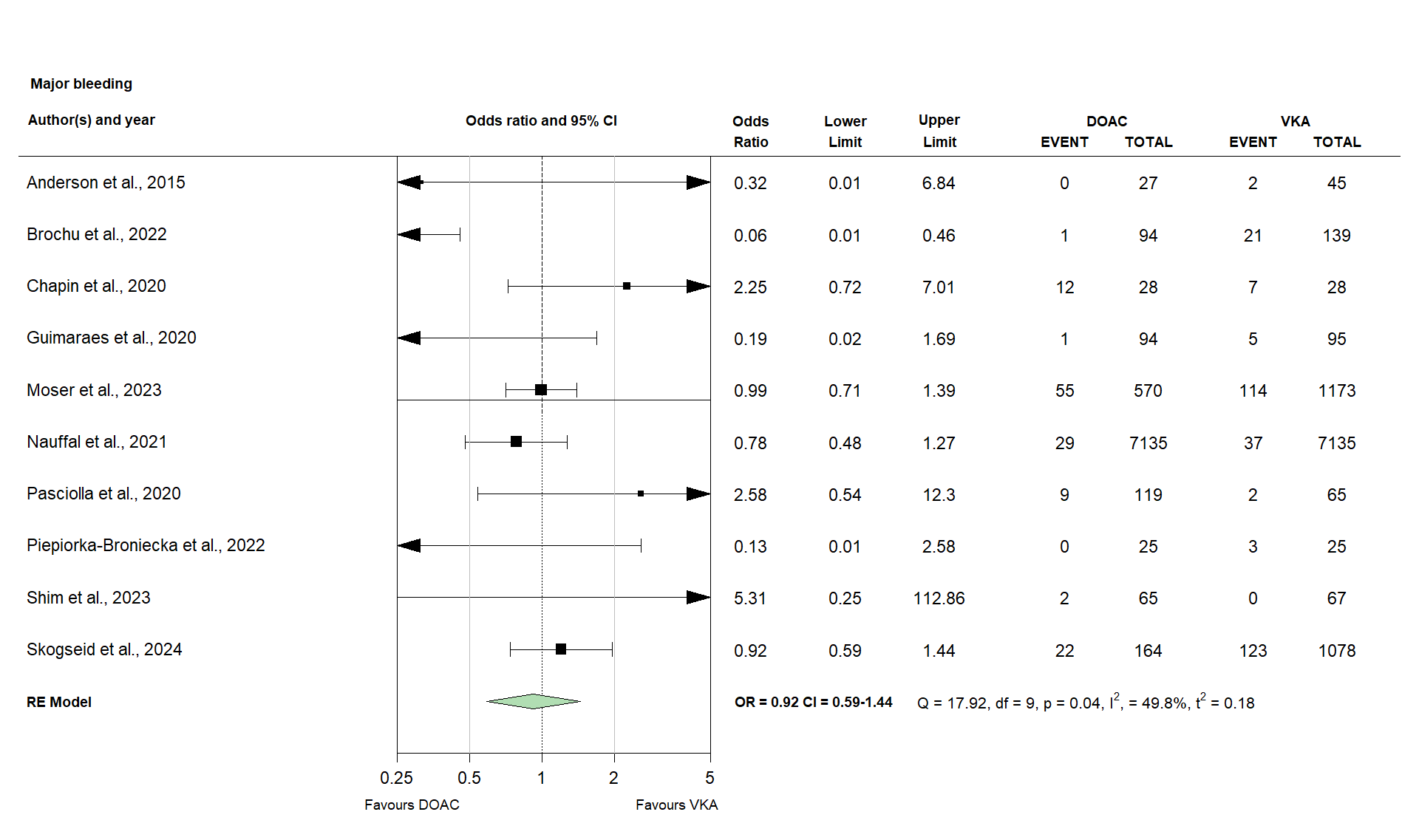
Supplementary Figure F6: Forest plot Major bleeding up to 12 months after anticoagulation initiation. RE: Random effects. Q: Q statistic for effect size variability assessment. Df: Degrees of freedom. I^2^: Extent of Heterogeneity. t^2^: Between study variance estimate.


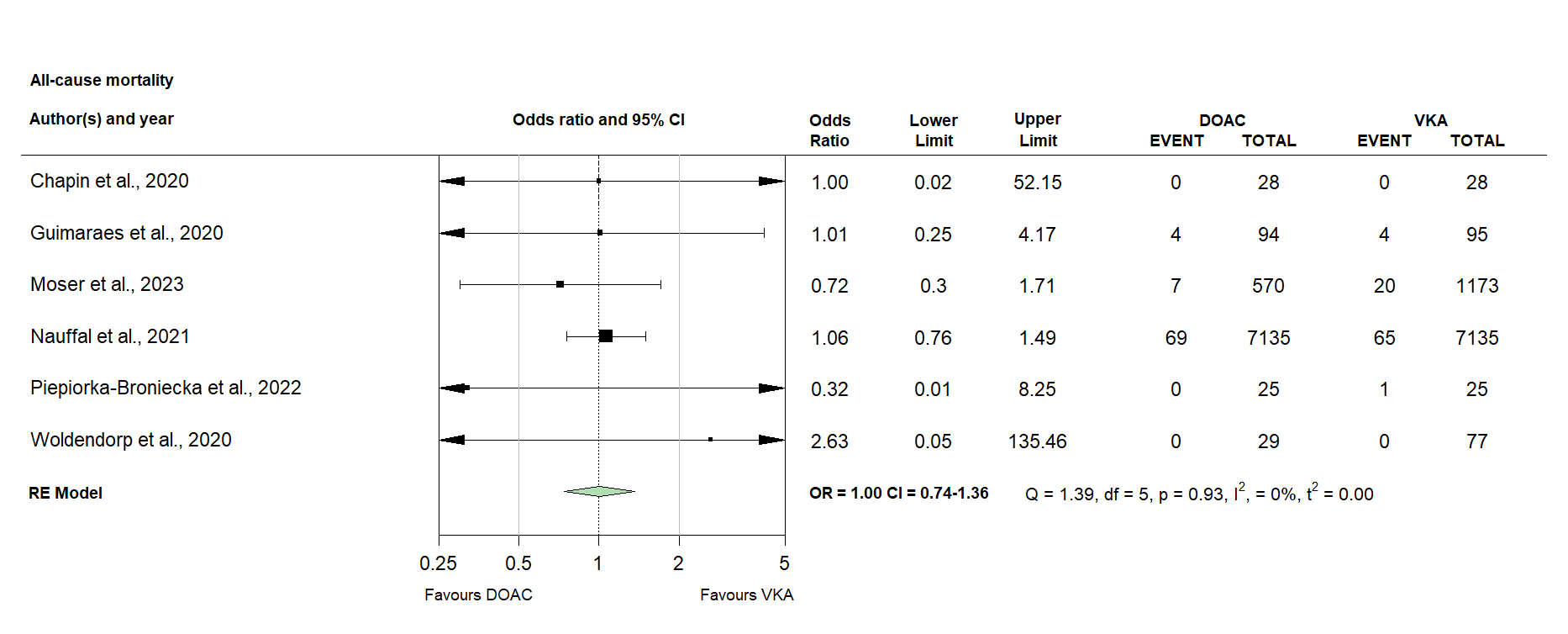


Supplementary Figure F7: Forest plot All-cause mortality up to 12 months after anticoagulation initiation. RE: Random effects. RE: Random effects. Q: Q statistic for effect size variability assessment. Df: Degrees of freedom. I^2^: Extent of Heterogeneity. t^2^: Between study variance estimate.

#### **
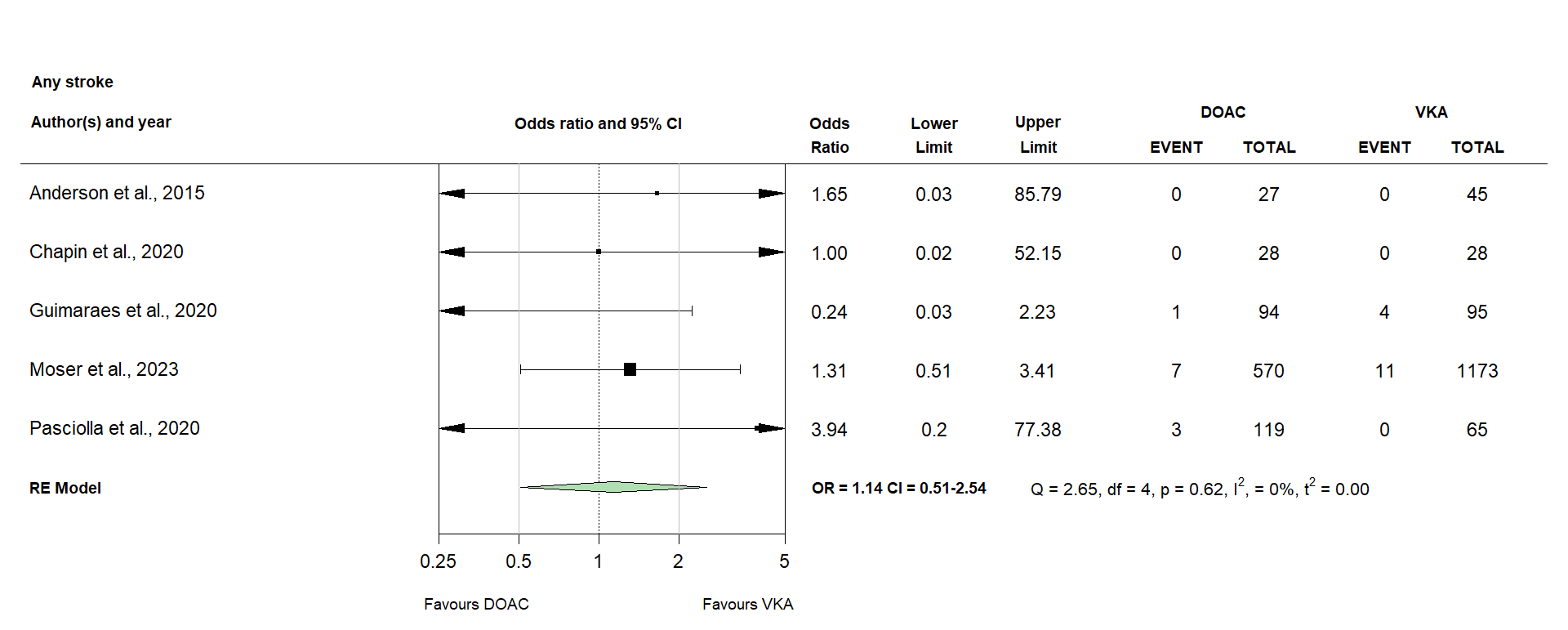
** Supplementary Figure F8: Forest plot Any stroke up to 12 months after anticoagulation initiation. RE: Random effects. Q: Q statistic for effect size variability assessment. Df: Degrees of freedom. I^2^: Extent of Heterogeneity. t^2^: Between study variance estimate.

##

## Supplementary Appendix F.4 Subgroup analyses


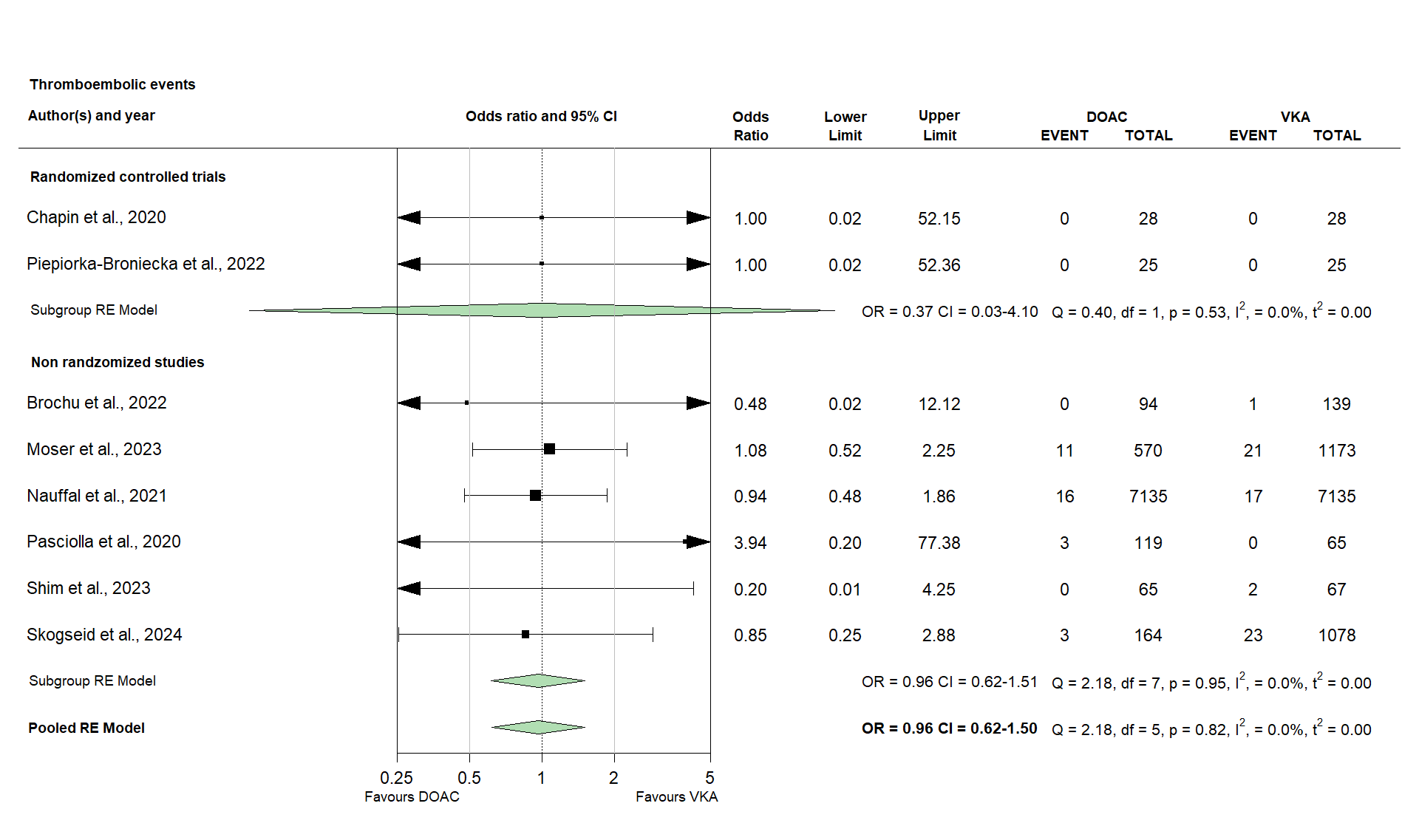


#### Supplementary Figure F9: Thromboembolic events per study design up to 6 months after anticoagulation initiation. RE: Random effects. Q: Q statistic for effect size variability assessment. Df: Degrees of freedom. I^2^: Extent of Heterogeneity. t^2^: Between study variance estimate.


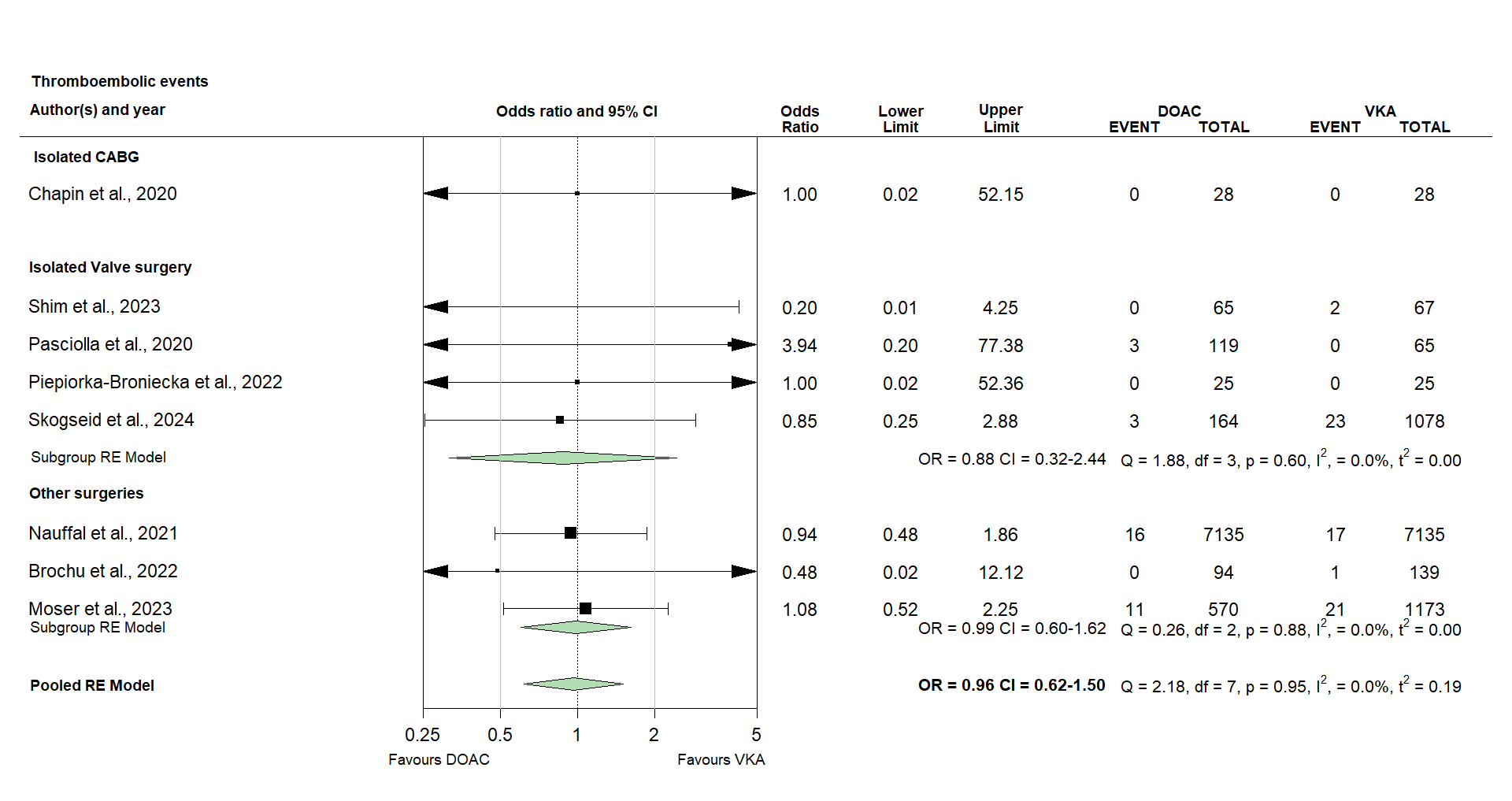
Supplementary Figure F10: Thromboembolic events per surgery type up to 6 months after anticoagulation initiation. RE: Random effects. Q: Q statistic for effect size variability assessment. Df: Degrees of freedom. I^2^: Extent of Heterogeneity. t^2^: Between study variance estimate.


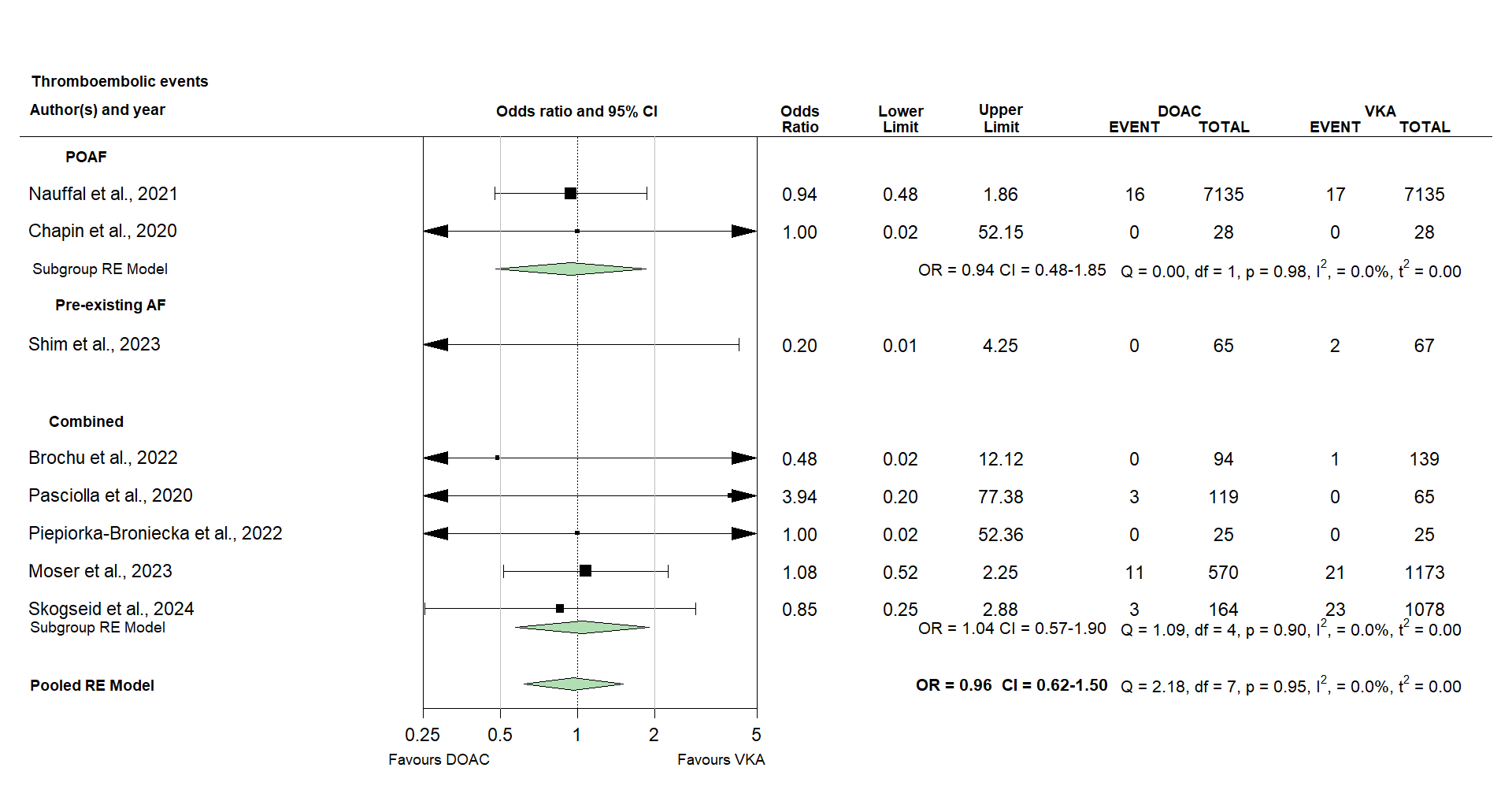


#### Supplementary Figure F11: Thromboembolic events per AF type up to 6 months after anticoagulation initiation RE: Random effects. Q: Q statistic for effect size variability assessment. Df: Degrees of freedom. I^2^: Extent of Heterogeneity. t^2^: Between study variance estimate.


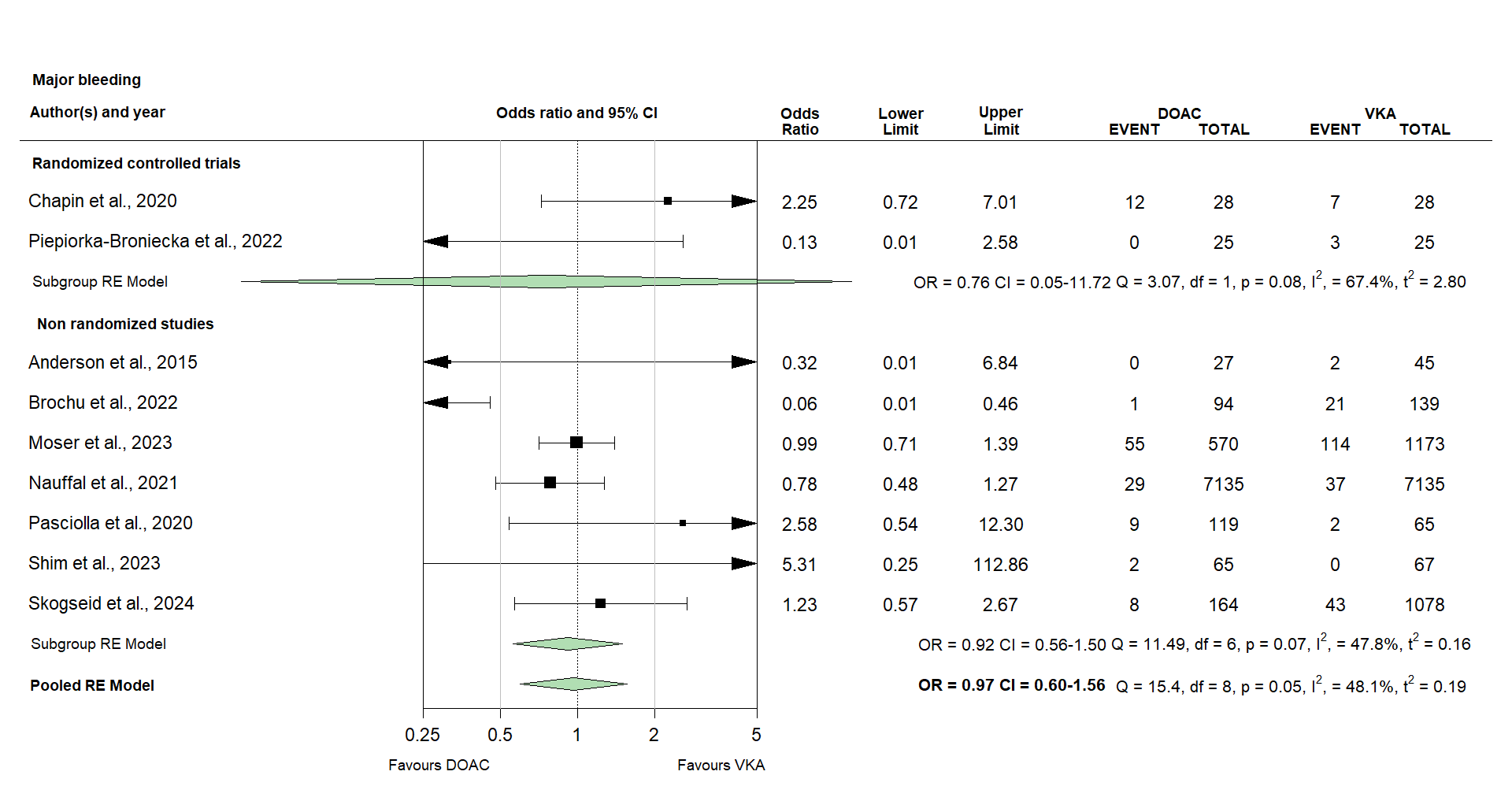


#### Supplementary Figure F12: Major bleeding per study design up to 6 months after anticoagulation initiation RE: Random effects. Q: Q statistic for effect size variability assessment. Df: Degrees of freedom. I^2^: Extent of Heterogeneity. t^2^: Between study variance estimate.


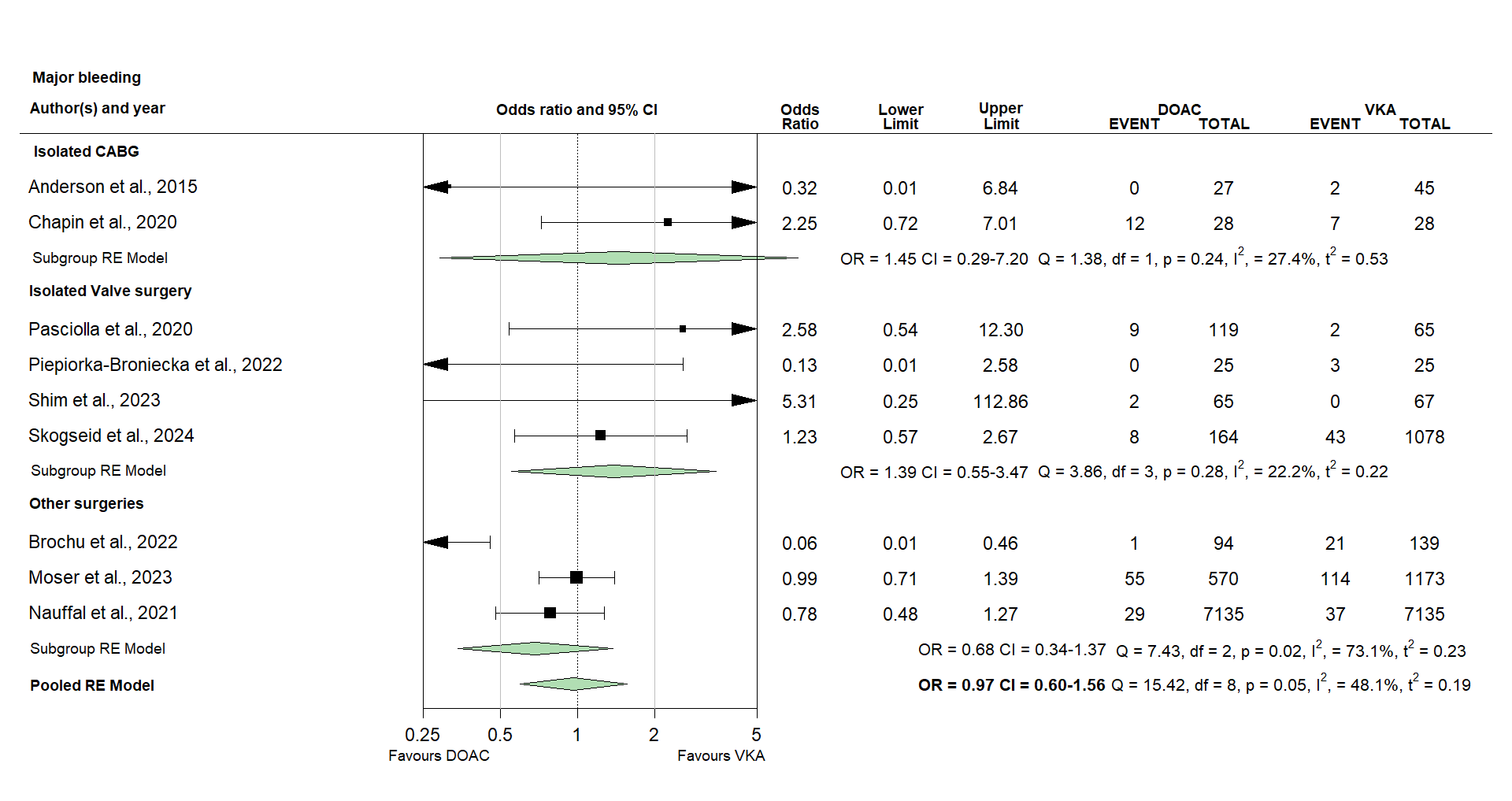


Supplementary Figure F13: Major bleeding per surgery type up to 6 months after anticoagulation initiation RE: Random effects. Q: Q statistic for effect size variability assessment. Df: Degrees of freedom. I^2^: Extent of Heterogeneity. t^2^: Between study variance estimate.

####
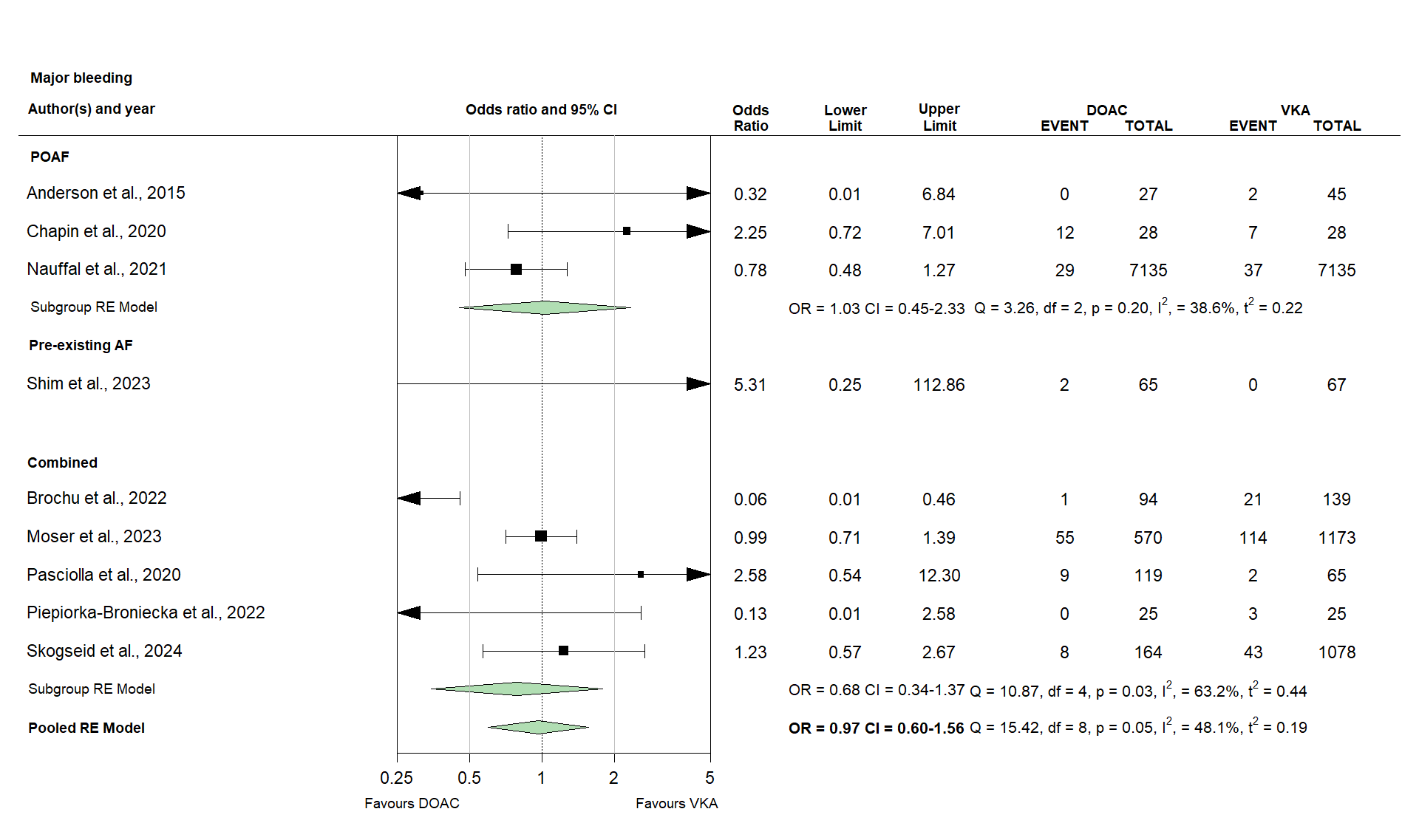
Supplementary Figure F14: Major bleeding per surgery type up to 6 months after anticoagulation initiation RE: Random effects. Q: Q statistic for effect size variability assessment. Df: Degrees of freedom. I^2^: Extent of Heterogeneity. t^2^: Between study variance estimate.

##
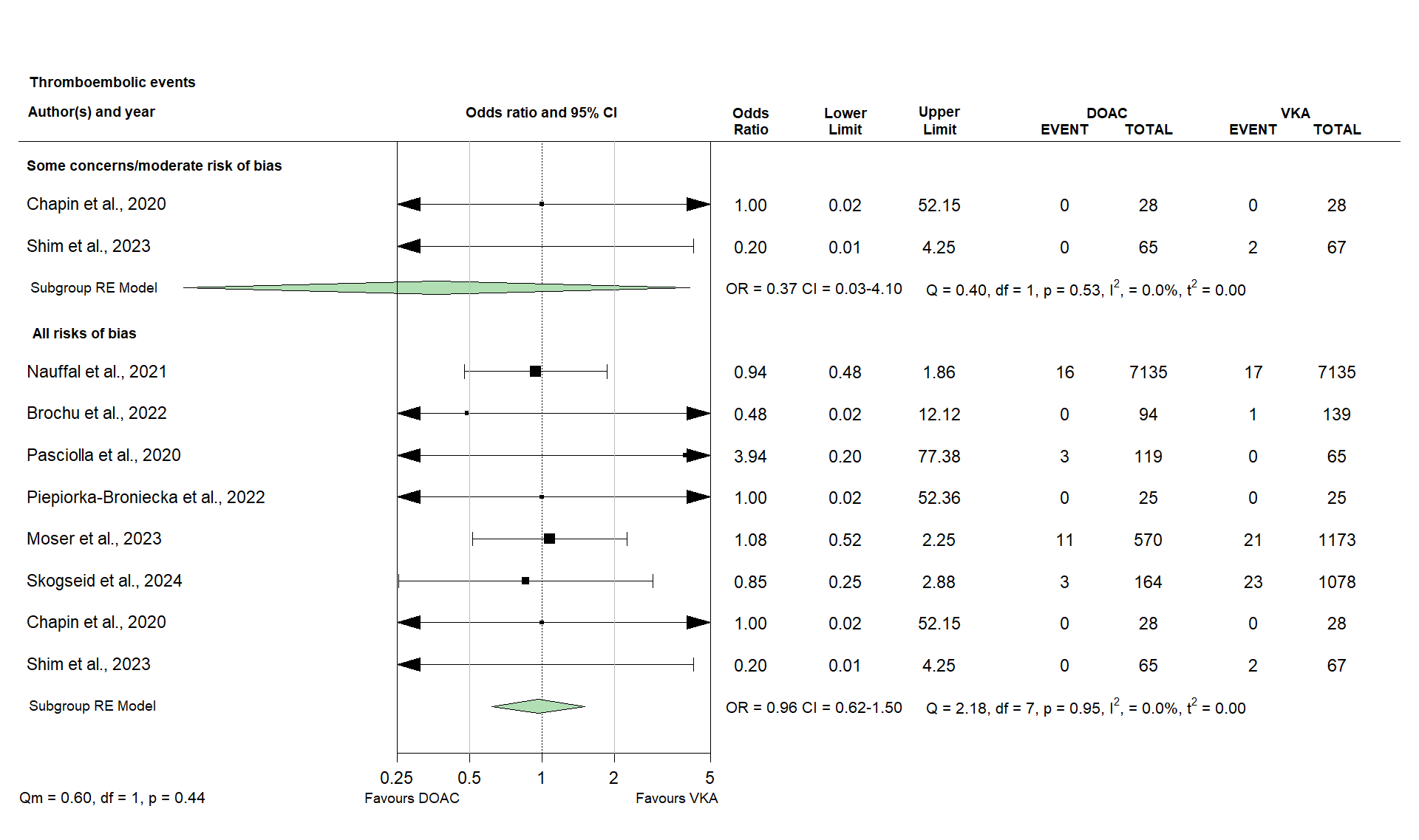
Supplementary Appendix F.5 Sensitivity analyses

#### Supplementary Figure F15: Thromboembolic events, sensitivity analysis some concerns / moderate risk of bias and all risks of bias. RE: Random effects. Q: Q statistic for effect size variability assessment. Df: Degrees of freedom. I2: Extent of Heterogeneity. t2: Between study variance estimate.


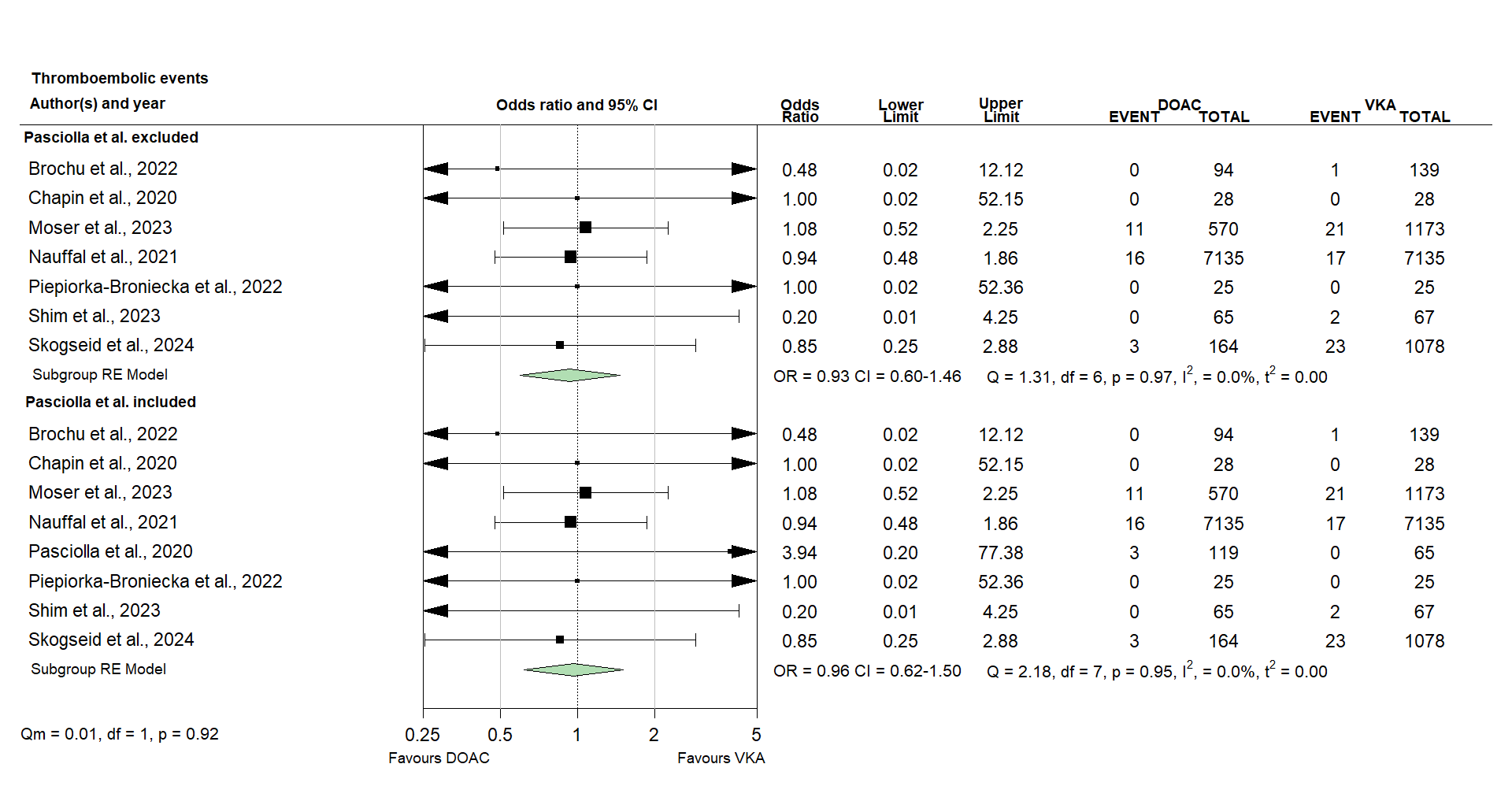

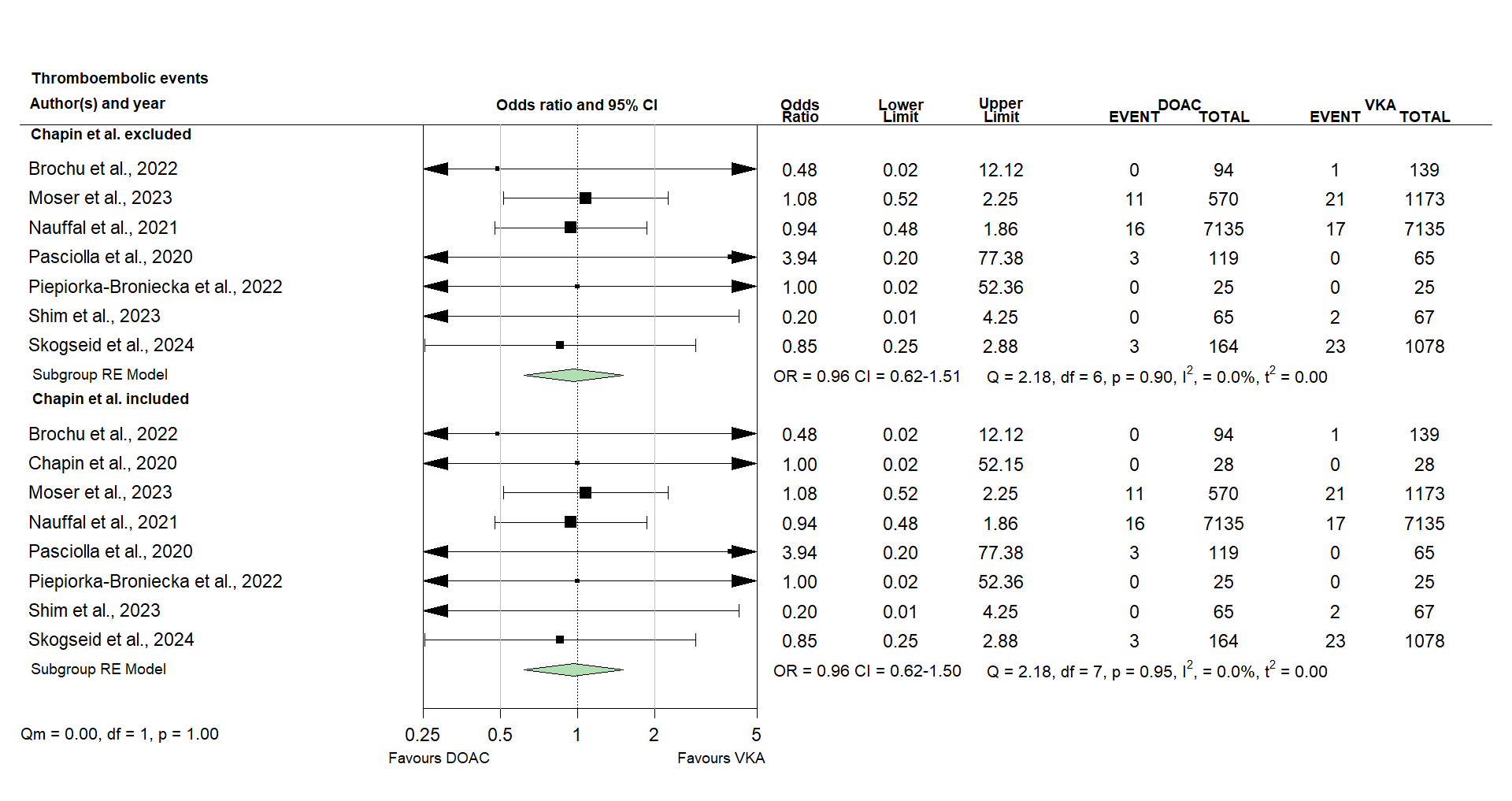
Supplementary Figure F16: Thromboembolic events, sensitivity analysis analysis inclusion and exclusion of the study by Chapin et al. RE: Random effects. Q: Q statistic for effect size variability assessment. Df: Degrees of freedom. I2: Extent of Heterogeneity. t2: Between study variance estimate.

Supplementary Figure F17: Thromboembolic events, sensitivity analysis inclusion and exclusion of the study by Pasciolla et al. Q: Q statistic for effect size variability assessment. Df: Degrees of freedom. I2: Extent of Heterogeneity. t2: Between study variance estimate.


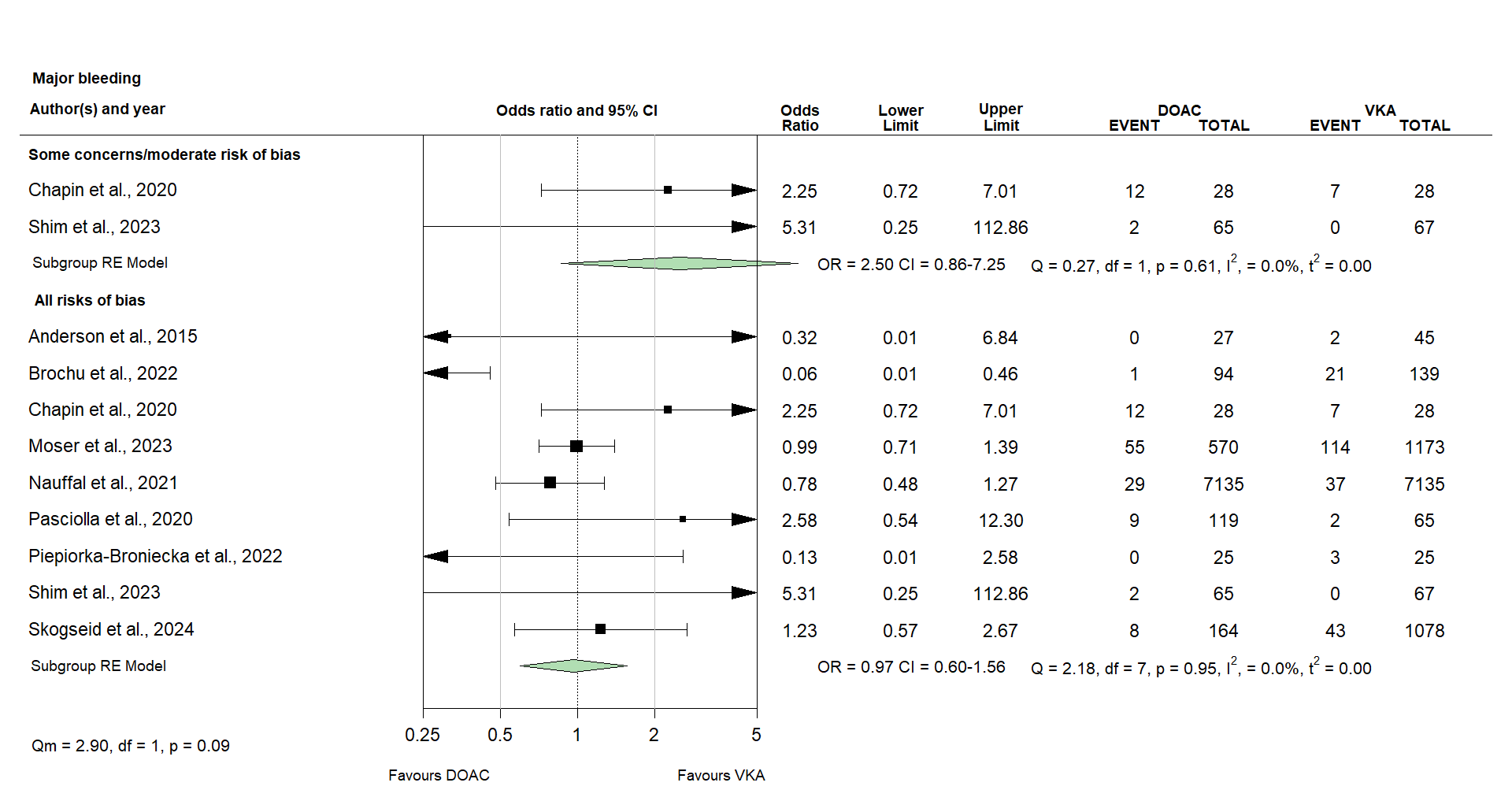
Supplementary Figure F18: Major bleeding, sensitivity analysis some concerns / moderate risk of bias and all risks of bias.

. RE: Random effects. Q: Q statistic for effect size variability assessment. Df: Degrees of freedom. I2: Extent of Heterogeneity. t2: Between study variance estimate.


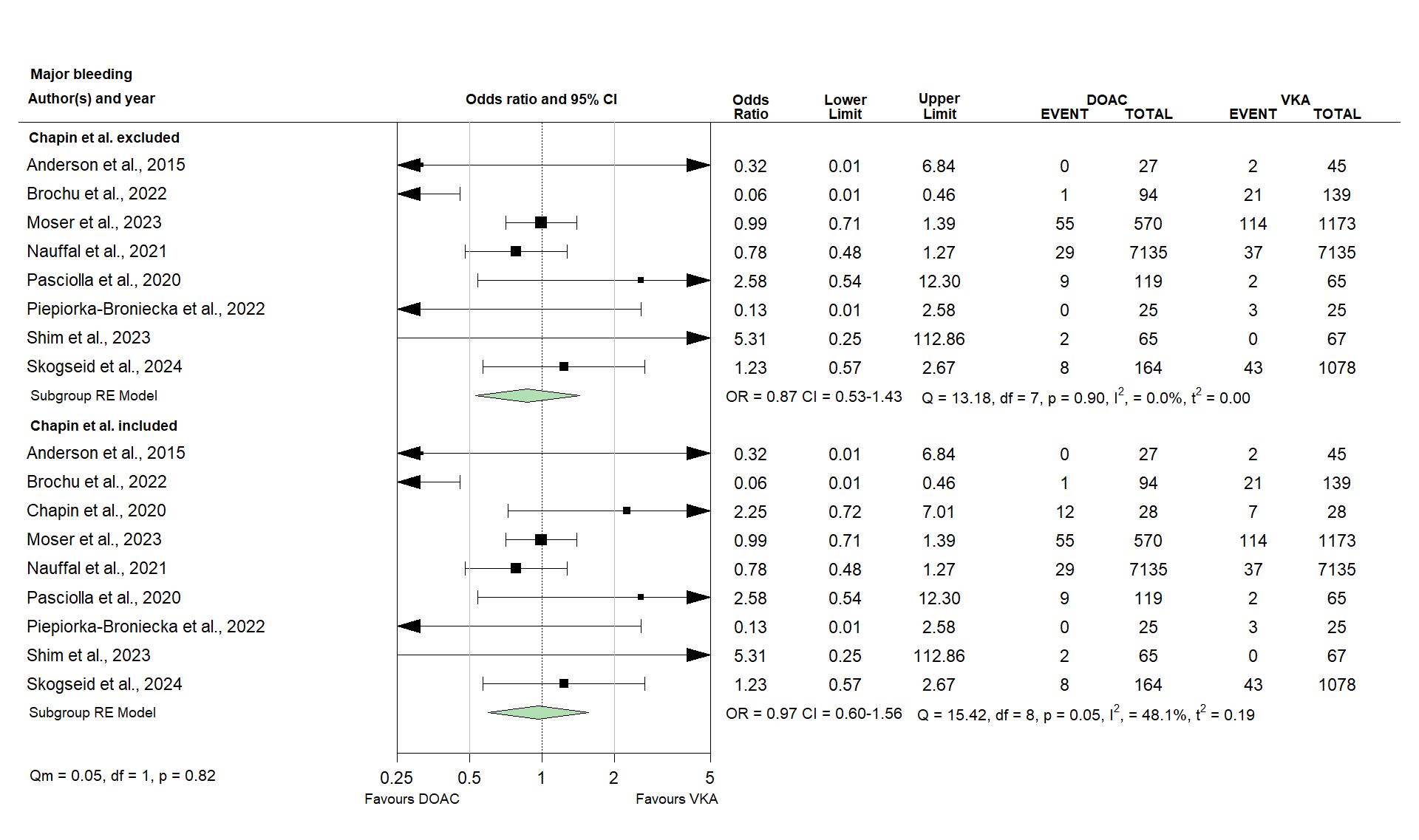


Supplementary Figure F19: Major bleeding, sensitivity analysis inclusion and exclusion of the study by Chapin et al. RE: Random effects. Q: Q statistic for effect size variability assessment. Df: Degrees of freedom. I2: Extent of Heterogeneity. t2: Between study variance estimate.


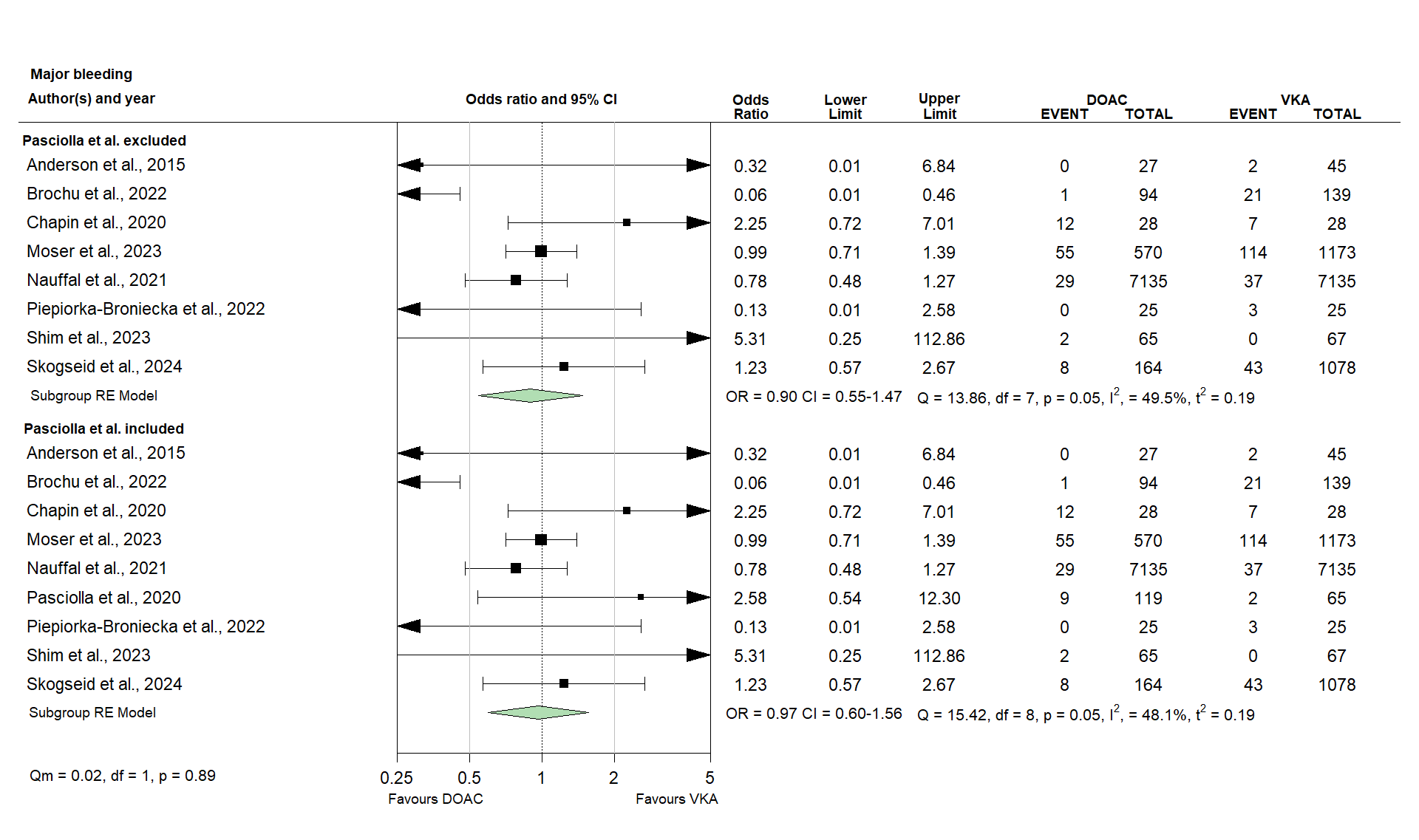


Supplementary Figure F20: Major bleeding, sensitivity analysis inclusion and exclusion of the study by Pasciolla et al. RE: Random effects. Q: Q statistic for effect size variability assessment. Df: Degrees of freedom. I2: Extent of Heterogeneity. t2: Between study variance estimate.

### Supplementary Appendix F.5 Meta-regression

### Supplementary Table F2:

| Outcome | Variable | n | Coefficient (CI) | P value | R^2^ | Tau^2^ | Qm | P value of Qm |
| --- | --- | --- | --- | --- | --- | --- | --- | --- |
| Thromboembolic events | Study Design  NRS  RCT | 6  2 | -  -0.0367(-2.7964 - 2.8698) | -  0.9797 | 0% | 0 | 0.0006 | 0.9797 |
|  | AF type  Combined  POAF  Pre-existing AF | 5  2  1 | -  -0.1018 (-1.0025 - 0.7990)  -1.6522 (-4.7658 - 1.4614) | -  0.8248  0.2983 | 0% | 0 | 1.0897 | 0.5799 |
|  | Surgery type  Other  Isolated CABG  Isolated Valve | 3  1  4 | -  0.0143 (-3.9707 - 3.9994)  -0.1150 (-1.2486 – 1.0185) | -  0.9944  0.8424 | 0% | 0 | 0.0399 | 0.9803 |
| Major bleeding | Study Design  NRS  RCT | 7  2 | -  0.4004 (-1.0201 - 1.8210) | 0.5806 | 0% | 0.2093 | 0.3052 | 0.5806 |
|  | AF type  Combined  POAF  Pre-existing AF | 5  3  1 | -  0.2620 (-0.9889 - 1.5129)  1.8894 (-1.4877 - 5.2666) | -  0.6814  0.2728 | 0% | 0.3807 | 1.2679 | 0.5305 |
|  | Surgery type  Other  Isolated CABG  Isolated Valve | 3  2  4 | -  0.8494 (-0.6721 – 2.3709)  0.7097 (-0.4561 – 1.8755) | -  0.2739  0.2328 | 0% | 0.2393 | 2.0544 | 0.3580 |
| Any Stroke | Study Design  NRS  RCT | 3  1 | -  -0.3810 (-4.4329 – 3.6709) | -  0.8538 | 0% | 0 | 0.0340 | 0.8538 |
|  | AF type  Combined  POAF | 2  2 | -  -0.1224 (-3.601 – 2.8153) | -  0.9349 | 0% | 0 | 0.0067 | 0.9349 |
|  | Surgery type  Other  Isolated CABG  Isolated Valve | 1  2  1 | -  -0.0205 (-2.9725 – 2.9315)  1.0974 (-2.0299 – 4.2228) | -  0.9891  0.4916 | 0% | 0 | 0.4797 | 0.7867 |

### Supplementary Table F3:

| Outcome | Variable | n | Coefficient (CI) | P value | R^2^ | Tau^2^ | Qm | P value of Qm |
| --- | --- | --- | --- | --- | --- | --- | --- | --- |
| All-cause mortality | Study Design  NRS  RCT | 3  2 | -  -0.6937 (-3.2235 – 1.8360) | -  0.5909 | 0% | 0 | 0.2889 | 0.5909 |
|  | AF type  Combined  POAF | 3  2 | -  0.3880 (-0.4985 – 1.2745) | -  0.3910 | 0% | 0 | 0.7359 | 0.3910 |
|  | Surgery type  Other  Isolated CABG  Isolated Valve | 2  2  1 | -  0.4766 (-2.3333 – 3.2865)  -1.1463 (-4.4100 – 2.1174) | -  0.7396  0.4912 | 0% | 0 | 0.5895 | 0.7447 |
| Admission duration | Study Design  NRS  RCT | 3  1 | -  1.4991 (-1.4349 – 4.4330) | -  0.3166 | 0% | 0.3935 | 1.0029 | 0.3166 |
|  | AF type  Combined  POAF | 2  2 | -  1.0169 (0.3143 – 1.7195) | -  0.0046* | 100% | 0 | 8.0487 | 0.0046 |
|  | Surgery type  Other  Isolated CABG | 2  2 | -  0.6866 (-1.8727 – 3.2453) | -  0.5989 | 0 | 0.4319 | 0.2766 | 0.5989 |

### Supplementary Appendix F.6 Funnel plots


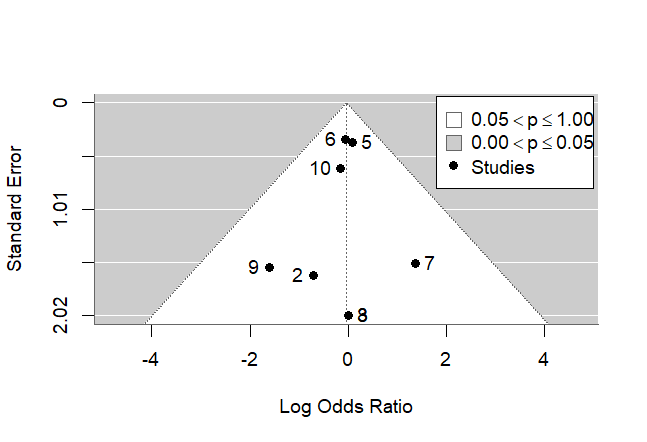


Supplementary Figure F21: Funnel plot thromboembolic events.


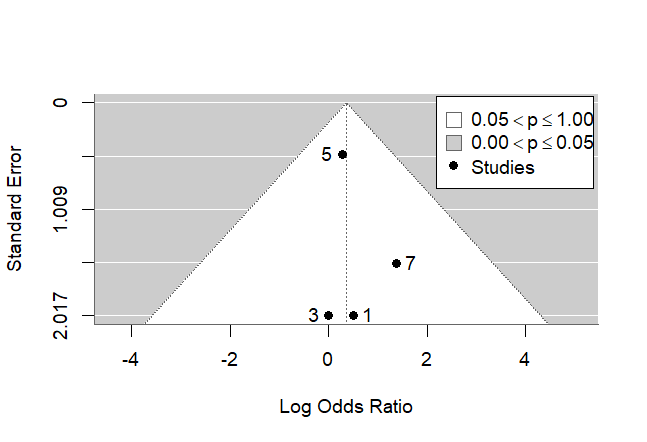


Supplementary Figure F2*4*: *Funnel plot any stroke.*

*
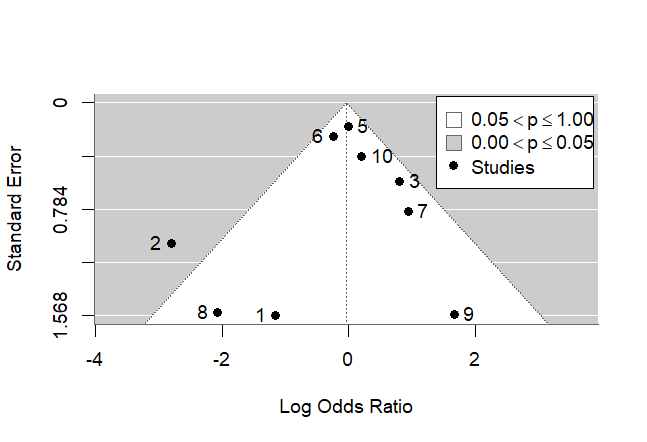
*Supplementary Figure F2*2*: *Funnel plot major bleeding*

*
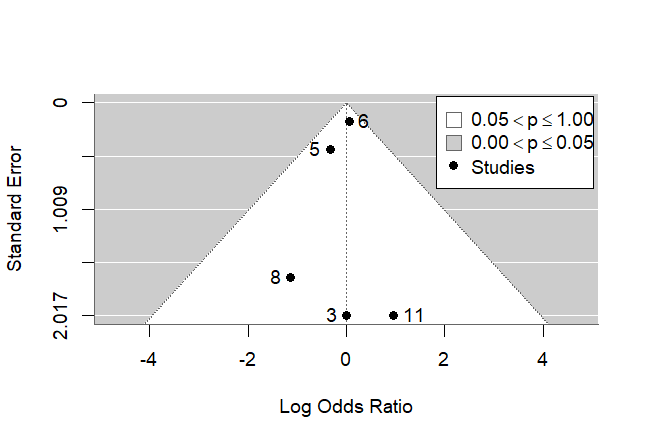
*

Supplementary Figure F2*3*: *Funnel plot all-cause mortality*


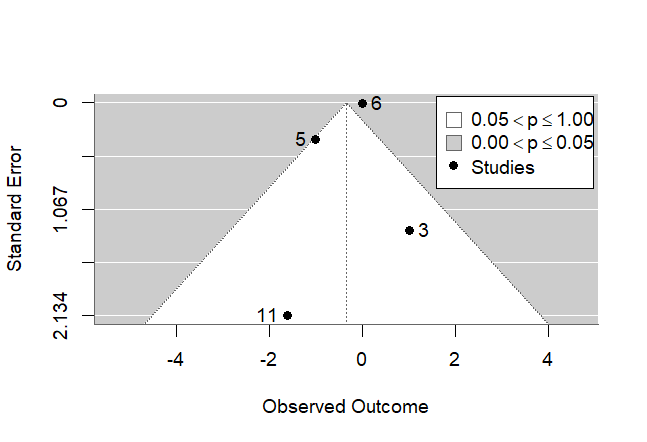


Supplementary Figure F2*5*: *Funnel plot admission duration*

*
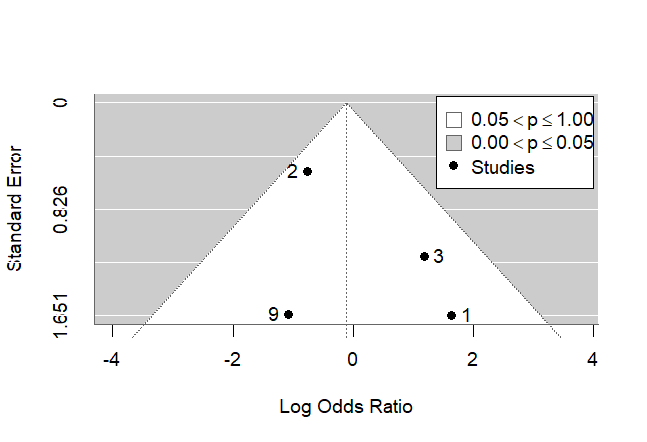
*

Supplementary Figure F26: *Funnel plot CRNM.*

*
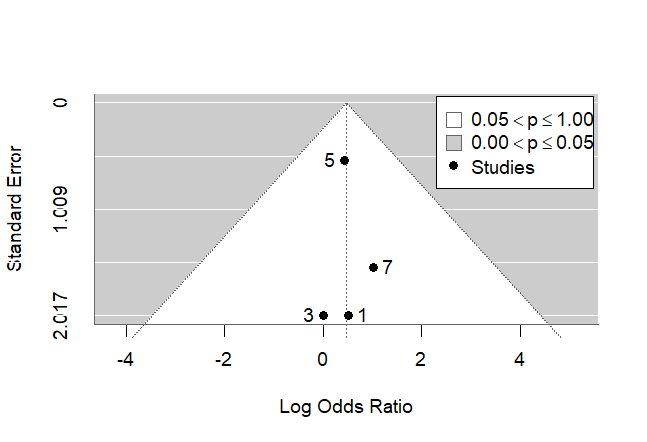
*

Supplementary Figure F2*7*: *Funnel plot Ischemic stroke*

*
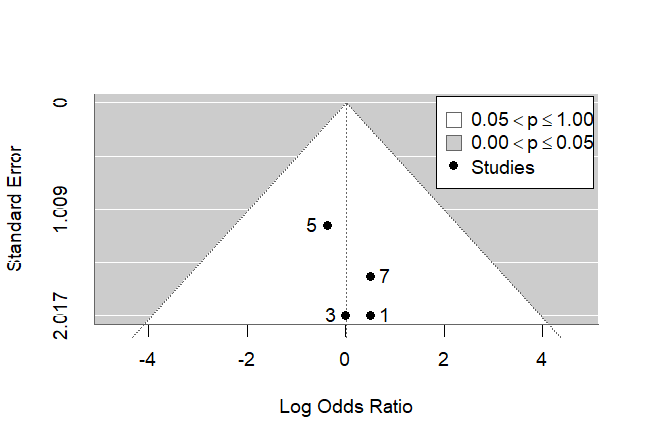
*Supplementary Figure F2*8*: *Funnel plot hemorrhagic stroke*

*
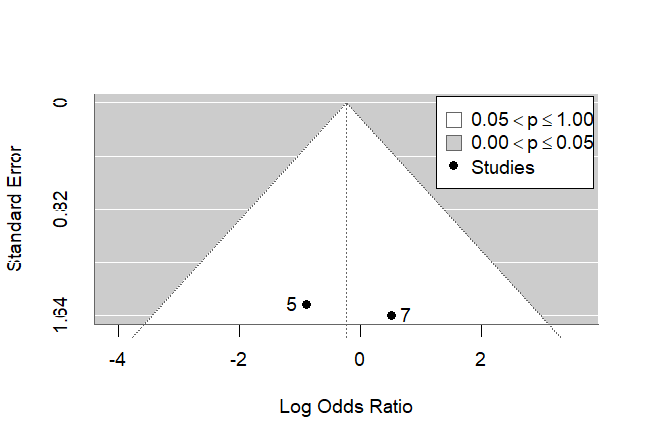
.*

Supplementary Figure F29: *Funnel plot systemic embolism*

# Supplementary References

1. Sterne JAC, Savovic J, Page MJ, Elbers RG, Blencowe NS, Boutron I, et al. RoB 2: a revised tool for assessing risk of bias in randomised trials. BMJ. 2019;366:l4898.

2. Sterne JA, Hernan MA, Reeves BC, Savovic J, Berkman ND, Viswanathan M, et al. ROBINS-I: a tool for assessing risk of bias in non-randomised studies of interventions. BMJ. 2016;355:i4919.

3. de Vries TAC, Hirsh J, Xu K, Mallick I, Bhagirath VC, Eikelboom JW, et al. Apixaban for Stroke Prevention in Atrial Fibrillation: Why are Event Rates Higher in Clinical Practice than in Randomized Trials?-A Systematic Review. Thromb Haemost. 2020;120(9):1323-9.

4. Schünemann H BJ, Guyatt G, Oxman A, editors. GRADE handbook for grading quality of evidence and strength of recommendations. Available from: <https://guidelinedevelopment.org/handbook>.

5. Schunemann HJ, Cuello C, Akl EA, Mustafa RA, Meerpohl JJ, Thayer K, et al. GRADE guidelines: 18. How ROBINS-I and other tools to assess risk of bias in nonrandomized studies should be used to rate the certainty of a body of evidence. J Clin Epidemiol. 2019;111:105-14.

6. Schünemann HJ, Neumann I, Hultcrantz M, Brignardello-Petersen R, Zeng L, Murad MH, et al. GRADE guidance 35: update on rating imprecision for assessing contextualized certainty of evidence and making decisions. J Clin Epidemiol. 2022;150:225-42.

7. Zeng L, Brignardello-Petersen R, Hultcrantz M, Mustafa RA, Murad MH, Iorio A, et al. GRADE Guidance 34: update on rating imprecision using a minimally contextualized approach. J Clin Epidemiol. 2022;150:216-24.

8. Higgins J, Thomas J. Cochrane Handbook for Systematic Reviews of Interventions 2022. Available from: <https://training.cochrane.org/handbook/current>.
